# Forms of Capital, Social Change and the Weight of the Past: The Effective Agents of the Swiss Field of Power 1910–2015

**DOI:** 10.1177/00380385251322061

**Published:** 2025-03-13

**Authors:** Thierry Rossier, Jacob Aagaard Lunding

**Affiliations:** University of Lausanne, Switzerland and London School of Economics, UK; Copenhagen Business School, Denmark

**Keywords:** capital, elites, field of power, history, social change, Switzerland

## Abstract

In this article we delve into the elites’ evolving forms of power to study the relationship between social change and capital accumulation. Drawing on Pierre Bourdieu’s notion of the field of power and relying on the identification of the field’s effective agents in Switzerland, we investigate the changing relations among the most important forms of capital. We use prosopographical data spanning six historical periods from 1910 to 2015 and thanks to multiple correspondence analysis we uncover the changing structure of the field of power. We show the dominance of economic and organisational network powers throughout history. While both forms of power opposed before the Second World War, they could be accumulated together between the 1950s and the 1980s but opposed again in the recent period. The article contributes to ‘big picture’ sociology, offering historical accounts of broad social trends and provides evidence of a recent return to past inequality logics.

## Introduction: Forms of Power and Social Change

In 1938, Maurice Halbwachs (1938: 37; our translation) wrote:

there is a section of the population which determines the directions, the main objects of common activity, and which also manifests the tendencies common to all better than the others. This is the highest class, the richest, the one that exercises the most important functions. It can be said ruling not only because it possesses the most material, political and economic power and authority, but also because of its ways of thinking, which are imitated and inspire the lower classes.

Like Mills (1956), Halbwachs stressed the existence of a ruling class – or an elite – with a disproportionate amount of power over society. In his argument, this group not only rules over the population, but also determines the ‘directions’, ‘main objects’ and ‘tendencies’ for other groups. However, the process through which the elites establish which issues and social stakes are the most important is not easy to uncover. Elites are found in a plurality of sectors (Aron, 1950) and form those who own a form of capital with a transferable value granting them power across many social fields (Khan, 2012). They are engaged in the struggles of definition of the main source of power. In this sense, the process of attributing the value and exchange rate between forms of capital should be at the centre of elite studies. To understand the dynamics of this attribution process, it is necessary to acknowledge that forms of capital are not static (Bourdieu, 1986). The accumulation of the main forms of capital has shaped current and former elite constellations, who in turn have shaped current and former societies (Savage and Nichols, 2018). As the elites are agents of social change and as accumulation needs to be considered over century-long time periods (Toft, 2024), we focus on struggles over forms of capital in the *longue durée*.

In this article, we ask two sets of research questions. First, what is the relationship between elites’ various forms of power? How do elites define the value and exchange rate between forms of capital? Which forms of capital are the most efficient in a given national context? Second, how do these dynamics evolve in the *longue durée*? How do forms of capital consolidate and erode? In summary, what is the relationship between social change and capital accumulation? To answer them, we rely on the notion of the *field of power* (Bourdieu, 1996b), where the dominant compete for the definition of the main societal power. To solve the problems of the field’s boundary circumscription and comparability over time we have found, in a former step, the field’s ‘effective agents’ – that is, the individuals with enough power to (re)define the field’s specific capital (Bourdieu, 2005; Lunding et al., 2021) – through the identification of the core of an elite network of the most influential institutions (Ellersgaard and Larsen, 2023; Larsen and Ellersgaard, 2017; Rossier and Ellersgaard, 2023; Rossier et al., 2022). We focus on this group’s structure to understand the relations among society’s most important forms of capital. As social inequality has been on similar levels in the last decades as during the ‘age of empire’ (Hobsbawm, 1987; Piketty, 2014) and as present structures carry the ‘weight of the past’ (Savage, 2021), we study social change and capital accumulation from the early 20th century onwards by understanding how power is structured among effective agents at different periods. In doing so, we follow Savage’s (2023) call for reviving ‘big picture’ sociology to offer historical accounts of broad social trends over a period of a century.

We focus on the Swiss case with its specificities, such as the absence of elite schools and universities, no unified cultural elite and only few symbolic credentials awarded by the state. To study the field’s changing forms of power, we rely upon six historical periods of effective agents (1910, 1937, 1957, 1980, 2000 and 2015),^1^ for a total of 731 individuals. We use specific multiple correspondence analysis (MCA) with variables on inherited capital and elite seniority, social and family background, and economic, cultural, social, symbolic and organisational capital to explore the evolving structure of forms of capital during 105 years.

In the conceptual section, we define the main forms of capital in the field of power and their transformations, then zoom in on the Swiss case. In the methodological section, we present how we identified the effective agents and our indicators. In the empirical section, we analyse the evolving structure of the field of power. We show that economic and organisational network powers constituted the most dominant resources throughout history. Before 1945, wealth opposed organisational network connections, while between the 1950s and the 1980s economic and organisational network powers could be accumulated together. Finally, since the 1990s, economic capital again opposes social and organisational capital, implying a return to past logics where wealthy capitalists and their representatives are as disconnected from organisational and state powers now as they were before the Second World War. In the concluding section, we answer our research questions and elaborate on the implications for research on social change.

## Theory: Fields of Power in a Historical Perspective

### Forms of Capital in the Field of Power

Within the complex system of fields – that is, relatively autonomous social spaces defined by their object of dispute (Bourdieu, 1996a) – the *field of power* is the field where the dominant are involved in conflicts and internal hierarchies. Its specific capital is a ‘capital conferring power over capital’ (Bourdieu, 2020: 34). Its owners, the *effective agents* (Bourdieu, 2005), define *effectively* the ‘value’ and ‘exchange rate’ between different forms of capital (Bourdieu, 1996b). To highlight the most effective forms of power, one needs to identify this group and uncover its relational structure (Lunding et al., 2021).

There are as many forms of capital – defined as resources that are part of systemic processes of advantage garnering (Bourdieu, 1986; Savage et al., 2005) – as there are fields (Bourdieu, 2020). Four forms of capital are effective in *any* social space. *Economic capital* relies on material resources. It has an *established* form, often inherited and related to the ownership of the means of production, and a *delegated* form, owned by company managers (Bourdieu and De Saint Martin, 1978). *Cultural capital*, in its embodied, objectified and institutionalised forms, relates to the advantages linked to culture and its effects (Bourdieu, 1979). *Social capital* is linked to possession of a network of relationships of mutual acquaintance or recognition (Bourdieu, 1980). Its volume partly depends on one’s multipositionality; that is, the positions held simultaneously in different fields (Boltanski, 1973). *Symbolic capital* corresponds to any capital in whatever form insofar as it is represented in a relationship of (mis)recognition (Bourdieu, 1986). Other resources combine with those forms of capital. *Organisational capital* relates to credentials provided by the linked organisations in affiliation networks, the position in those organisations and other career properties (Bourdieu, 2005; Ellersgaard et al., 2013, 2019). The French field of power in the 1970s was structured by two main principles. The first one implied a *chiastic structure* between holders of economic and cultural capital. The second principle related to the *global volume* of capital (Bourdieu, 1996b). Nevertheless, social spaces are further shaped by a third principle, that is *time* and its effects (Bourdieu, 1984).

### The Field of Power and Its Transformations

Field structures are evolving and, although inertia mechanisms usually prevent them from rapid structural changes, internal struggles lead to the redefinition of the main effective forms of capital (Bourdieu, 1996a). Forms of capital take time to be accumulated (Bourdieu, 1986) and accumulation needs to be considered over long time periods – over several generations or at least a century (Nichols and Savage, 2017; Savage and Nichols, 2018).

At the beginning of the 20th century, elites in the field of power were involved in strong processes of *family-based reproduction* (Bourdieu, 1996b). Business elites inherited economic assets allowing them access to the control of economic institutions (Fellman, 2014; Kansikas, 2016; O’Brien, 2024). Wealth and other forms of economic capital were extremely concentrated in the hands of a small number of individuals (Alvaredo et al., 2013). The importance of *educational-based reproduction* intensified afterwards, as access to dominant positions was increasingly subordinated to the ownership of educational credentials (Bourdieu, 1996b; Friedman and Reeves, 2020; Reeves et al., 2017; Worth et al., 2023). These dynamics led the ownership of economic and cultural capital to be the central structuring forms of power until the 1970s.

Since the 1980s, the chiastic structuration lost in importance. Neo-liberalism subjugated politics and culture to the goals of business (Bourdieu, 1999; Denord and Lagneau-Ymonet, 2016), and economic and cultural capital were progressively accumulated together (Bourdieu, 2019). Due to a growing incursion of economic capital into social life, many fields lost their autonomy (Savage, 2021). Wealthy inheritors became increasingly capable of converting their economic advantages into dominant positions (Toft and Friedman, 2020). This trend illustrates a return to past logics, where wealth and other forms of economic capital have been as concentrated in the recent period as they were before the First World War (Piketty, 2014). Scholars have studied more recent national iterations of the field of power. While each time the field presents national specificities, *inclusion into the economic order* (i.e. the volume of economic capital) is one of the main forms of distinction in France (Denord et al., 2018), Norway (Denord et al., 2011; Flemmen, 2012; Hjellbrekke et al., 2007) and Denmark (Lunding et al., 2021). Business elites and holders of economic capital used to own many personal and organisational connections in various sectors. However, corporate networks fragmented during the last decades (Mizruchi, 2013) and the elite fractions rich in economic capital do not own as much social and organisational capital as they used to.

### The Evolutions of the Swiss Field of Power

Several features of Switzerland make it a particularly relevant case to study elite power. First, business associations and large (multinational) companies strongly influence the coordination of the Swiss economy (David et al., 2015; Mach et al., 2016). Family-based transmission of *economic capital* has been particularly important and for a long time prevented outsiders from gaining control over Swiss companies (Ginalski, 2013, 2021). Second, the elites are involved in vast individual-organisation networks allowing them to coordinate across sectors and acquire *social* and *organisational capital* along their career (Bühlmann et al., 2012; Mach et al., 2007). Swiss elites meet in diverse organisational structures across sectors developing a strong multipositional profile (David et al., 2009; Eichenberger and Ginalski, 2017; Rebmann and Mach, 2013). The country’s small size allows for the concentration of elites in a small number of cities where their chances to meet and live close to one another are high (Benz et al., 2024b, 2025). Third, unlike France or the UK, the Swiss educational system features no elite schools or universities linking *cultural capital* to a class-specific habitus from the upper classes (Hartmann, 2010). More importance is granted to the discipline of university diplomas and doctoral titles, like in Germany (Hartmann, 2000). Related to the country’s multi-language configuration and the cultural proximity of big neighbouring countries, the influence of national cultural organisations is weak, resulting in a lowly unified cultural elite (Mach et al., 2024). Finally, unlike other more centralised European countries with a monarchy and/or a colonial history, the Swiss federal state does not award royal distinctions, nobility titles or national medals of merit. *Symbolic capital* is rather granted through the connections to prestigious and powerful organisations (especially the oldest ones), the belonging to the largest cities’ old patrician families through their last names or some other forms of public visibility, for example in the media (Benz et al., 2024a).

The history of Swiss elite integration is divided into three historical periods (Rossier and Ellersgaard, 2023; Rossier et al., 2022). In the first, from the end of the 19th century until the Second World War, the elites were consolidated around powerful banking and industrial firms and through family-based reproduction (Araujo et al., 2024; Strebel and Mach, 2023). The Swiss elite network core was mostly formed of business elites, occupying positions in multiple sectors. During the second period, from 1945 to the 1980s, the Swiss elites became even more integrated and the core became more diverse in terms of sectors (Bühlmann et al., 2017). In the last period, the elite network entered a fragmentation logic. The core became once again more composed of business elites, who were this time less connected and multipositional than before (Mach et al., 2021). We use this periodisation to assess social change in Switzerland, based upon the evolving configurations of forms of capital within the elite core, conceptualised as effective agents within the field of power. By focusing on elite biographies, families, generations and periods during 105 years, we uncover how elite constellations carry the weight of the past.

## Methods

### Finding the Effective Agents from the Swiss Field of Power

To identify effective agents within the Swiss field of power, we used data gathered through multiple sources in the comprehensive Swiss Elite Database^2^ that allowed us to include memberships in the most influential institutions in Swiss society. We relied on a list of names (n = 18,435) and organisational affiliations (n = 2174) from key sectors (companies, business associations, unions, politics, civil service, academia, state expertise, interest organisations and the military) at six benchmark years to build two-mode (i.e. individual-to-organisation) networks. As we could not possibly collect elite data for each year, we chose to focus on periods that were representative of different moments in Swiss history with an acceptable number of years in-between to assess the appearance of elite groups and generations. We focused on the following periods: before the First World War (1910), the interwar period (1937), the post-war period (1957), the period before (1980) and after (2000) financialisation processes, and the most recent years (2015).^3^ We transformed those networks into one-mode (elite-to-elite) networks tied by 319,045 connections in total and used a k-shell decomposition procedure (for more details, see Ellersgaard and Larsen, 2023; Larsen and Ellersgaard, 2017) to identify the central and cohesive ‘core’ in each elite network. Added up, those elite cores were formed in total of 731 individuals (Rossier and Ellersgaard, 2023; Rossier et al., 2022).^4^ The elite core is very similar to Mills’ (1956: 18) definition of the power elite as an ‘intricate set of overlapping cliques [that] share[s] decisions having at least national consequences’. We use this group to approximate the effective agents of the Swiss field of power (Denord et al., 2018; Lunding et al., 2021). Table 1 summarises the composition of the effective agents. They have always been dominated by business groups, especially during the first (1910, 1937) and the last (2000, 2015) periods, while during the peak integration period (1957, 1980), the effective agents were more diverse. This evolving composition that has been determined by society-wide social change processes has in turn a direct impact on the changing importance of forms of capital in society.^5^

**Table 1.** Composition (main affiliation sector) of the effective agents.

Sector	1910	1937	1957	1980	2000	2015
Companies	77% (58)	82% (84)	33% (69)	27% (53)	44% (42)	65% (32)
Business associations	3% (2)	6% (6)	21% (45)	24% (48)	31% (30)	14% (7)
Politics	20% (15)	10% (10)	24% (51)	16% (32)	4% (4)	6% (3)
Unions	0% (0)	1% (1)	8% (17)	9% (18)	11% (11)	2% (1)
Administration	0% (0)	0% (0)	8% (17)	19% (37)	5% (5)	0% (0)
Academia	0% (0)	2% (2)	6% (12)	5% (9)	4% (4)	12% (6)
Total	100% (75)	100% (103)	100% (211)	100% (197)	100% (96)	100% (49)

*Notes*: For example, in 1910 77% of effective agents were affiliated to companies as their main sector.

We relied upon a thorough prosopographical data collection (Lunding et al., 2020; Rossier, 2019). Our data on effective agents’ biographies, families and affiliations stem from a variety of sources, such as public archive collections, digitalised archival documents, newspapers, biographical dictionaries, genealogical websites, organisational reports, as well as social network analysis techniques, media coverage from a large list of newspapers and rankings of patronyms among a larger elite set.

### The Historical Geometry of Power

To investigate historically the Swiss field of power, we rely upon a thorough theoretically informed descriptive strategy (Savage, 2009, 2020, 2024) through MCA. MCA represents geometrically the structures of a set of *active* variables through different axes of opposition, where every variable modality and every individual has a numeric coordinate. We interpret the axes based upon the contributions of modalities and variables. Using *specific* MCA allows us to project some active modalities (e.g. missing values) as *passive* (Hjellbrekke, 2019; Le Roux and Rouanet, 2010). We run one MCA per year and compare the first axes of each analysis. The MCA spaces are formed of 32–34 variables (82–94 modalities) organised into seven variable blocks, whose composition evolves over time: *inherited capital and seniority* (2–4 variables); *social and family background* (4–6 variables); *cultural capital* (3–5 variables); *economic capital* (4–5 variables); *social capital* (7–8 variables); *symbolic capital* (four variables); *organisational capital* (five variables). Table 2 lists all the variables.^6^

**Table 2.** Variable blocks.

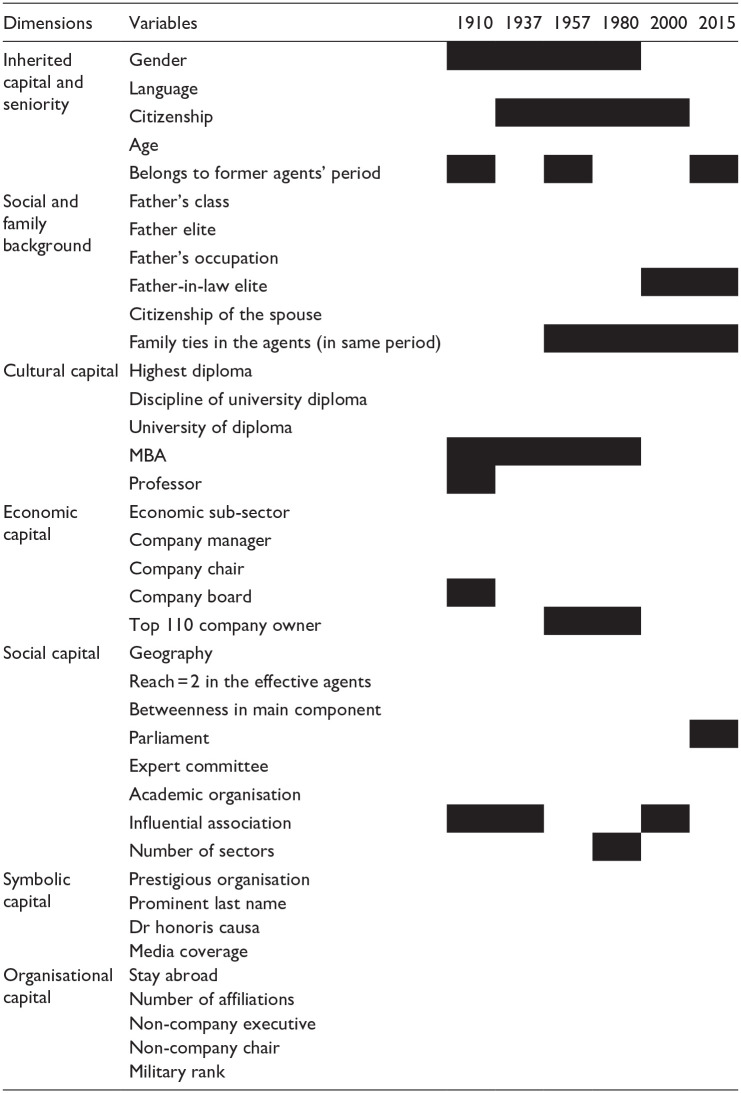

*Notes*: A black cell means that the variable was not used that year.

## Analysis: Evolving Forms of Capital in the Field of Power

**Figure 1.**
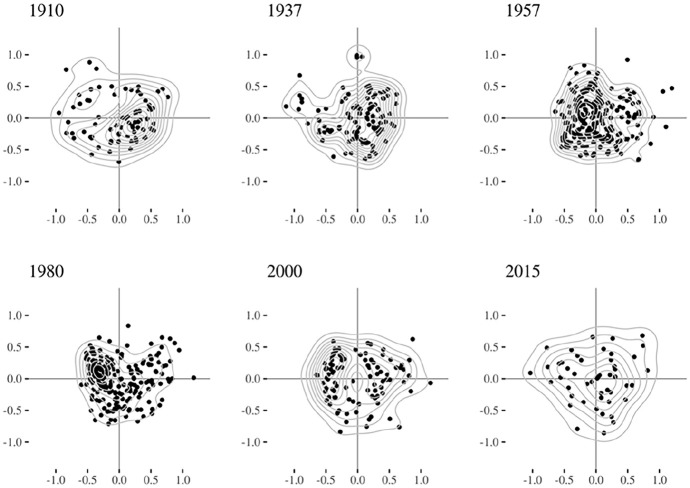
Individual clouds.

**Figure 2.**
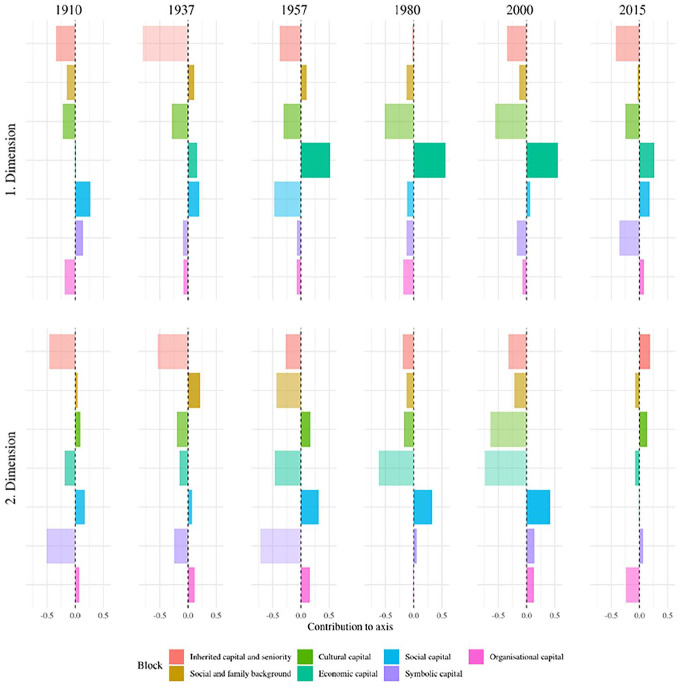
Importance of variable blocs in contributing to axes 1 and 2. *Notes*: The importance of a variable bloc to a given axis is given by the ratio between its relative contribution to the axis (see Le Roux and Rouanet, 2004) and its relative contribution to the total variance, $R=\frac{Ctr_{b}^{l}}{Ctr_{b}}$ , transformed to the range [−1,1] by $\frac{R-1}{R+1}$ . The value 0 then means that the contribution of the variable bloc to the axis corresponds to its overall contribution. The more positive the value the greater importance to the axis of a variable bloc relative to its overall weight. For example, in 1910, social capital’s relative contribution to axis 1 is higher than its relative contribution to the total variance.

**Figure 3.**
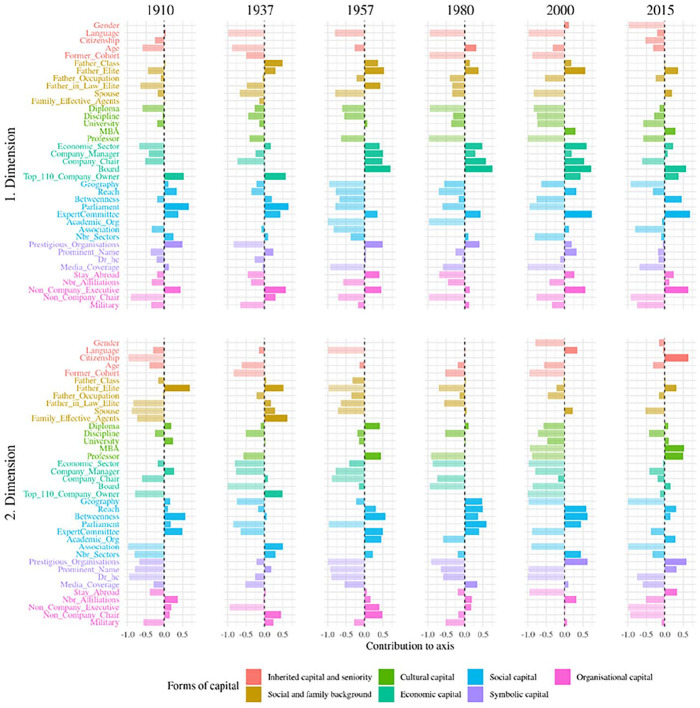
Importance of the variables in contributing to axes 1 and 2. *Notes*: The importance of a variable to a given axis is given by the ratio between its relative contribution to the axis (see Le Roux and Rouanet, 2004) and its relative contribution to the total variance, $R=\frac{Ctr_{b}^{l}}{Ctr_{b}}$ , transformed to the range [−1,1] by $\frac{R-1}{R+1}$ . The value 0 then means that the contribution of the variable to the axis corresponds to its overall contribution. The more positive the value the greater importance to the axis of a variable relative to its overall weight. For example, in 1910, for the ‘parliament’ variable, its relative contribution to axis 1 is higher than its relative contribution to the total variance.

We proceeded to six MCAs, using each time different or differently coded variables according to the period’s specificities, to uncover the main oppositions among effective agents at six historical benchmarks. Given the quantity of results, we only focus on the MCAs’ first two axes.^7^ Figure 1 displays the six individual clouds, Figure 2 the evolution of the contributions of the seven variable blocs and Figure 3 the importance of the contributions of the variables. Table 3 summarises qualitatively the main forms of capital along the two first axes. We describe the changing MCA axes according to the three historical periods identified in the history of Swiss elites.

**Table 3.** Summary of mains forms of capital, first two axes.

	Axis 1	Axis 2
1910	Inherited (established) economic capital vs. Social, organisational and symbolic capital λ = 46.8%	Inherited cultural capital (law professionals) vs. Social, organisational and (delegated) economic capital λ = 14.3%
1937	Inherited (established) economic capital vs. Social, organisational, cultural and symbolic capital λ = 36.4%	Volume of capital λ = 19.4%
1957	Volume of inherited (established and delegated) economic capital λ = 33.0%	Volume of social, organisational and cultural capital λ = 20.3%
1980	Volume of inherited (established and delegated) economic capital λ = 43.0%	Volume of social, organisational and symbolic capital λ = 15.0%
2000	Inherited (established and delegated) economic capital vs. Social and organisational capital λ = 45.7%	Volume of social, organisational and symbolic capital λ = 15.2%
2015	Inherited (established and delegated) economic capital vs. Social and organisational capital λ = 33.5%	Inherited (established) economic, social and symbolic capital vs. Cultural capital λ = 16.8%

*Notes*: λ denotes modified variance rates.

### Early 20th Century to the Second World War (1910, 1937): Organisational Network Connections vs. Inherited Wealth

During the first period, at times of huge wealth inequality and when elites were still consolidating their network integration – before the Second World War – economic and social capital were particularly contributing to the *first MCA axis*. Our results showed a similar opposition for 1910 and 1937. On the one hand, we found the individuals with a huge volume of social capital, in the form of network connections and sector number, and who sat on the most central political organisations. They also owned organisational resources, such as chairman positions. They inherited cultural capital, as their father was from the cultural fractions of the upper and upper-middle classes, and had himself a diploma in law. As they were involved in politics, they had an important coverage in the media. This group was therefore constituted by the most connected individuals. On the other side of the spectrum, we found owners of the largest Swiss companies, themselves inheritors of company owners or from another upper-class position, with a father who himself occupied an elite position, as well as other family ties in the elite. This form of economic capital was marked by extreme wealth and by an established (rather than delegated) form of economic resources. This axis represented the opposition between organisational network relations and inherited wealth.

The *second axis*, in 1910, opposed law professionals with political or organisational roles, themselves children of elite members with a professional background in law, to connected company managers and chairmen outside the business sector. In 1937, it corresponded to the volume of all forms of inherited and acquired (economic, cultural, social, symbolic, organisational) capital.

### From the Second World War to the 1980s (1957, 1980): Integration into the Economic Order and into Organisational Networks

In this period, when elites strongly integrated in a corporatist system of expert committees reuniting many societal sectors, economic capital nevertheless hugely contributed to the first axis, while social capital was strong on the second axis. On the *first axis*, delegated forms of economic capital were the most structuring, as we were not able to project top company ownership as an active variable (in 1957 it was too associated with other characteristics, and in 1980 the numbers were too low for the ‘Yes’ modality). We found, on one pole of the axis, the elites with an economic upper-class background and strong elite family ties, who sat on the board of historically prestigious companies, sometimes as chairman or CEO, as well as on many other sectors outside business. Top company owners were nevertheless among them.^8^ On the other pole, we found individuals with no economic positions whatsoever. This axis related to the volume of inherited economic capital, with mostly delegated – yet also established – forms of economic power; that is, what Denord et al. (2018: 287) called the ‘integration into the economic order’.

The *second axis* corresponded to the volume of social and organisational capital (reinforced by cultural capital in 1957, and symbolic capital in 1980). On the one hand, we found individuals that were very central in the affiliation network and who sat simultaneously on many sectors and affiliations, with top managerial positions and, on the other, individuals with none of such connections and positions.

### From the 1990s Onwards (2000, 2015): Organisational Network Connections vs. Inherited Wealth and Delegated Economic Power

During this period when the elite network started to fragment and wealth inequality was on the rise, we saw once more that economic capital indicators contributed disproportionately to the *first axis*. On one side of the axis, we found the elites who sometimes owned a top company or at least sat on the board of it, who went through an executive education and with a father who was a company owner and occupied an elite position himself. On the opposite side, we found the elites with large network connections in terms of centrality and ties to important organisations, occupying managerial roles in those institutions. This axis resembled the one during the first period, when organisational network connections opposed inherited wealth (but this time in conjunction with delegated forms of economic power).

In the case of 2000, the *second axis* was very similar to the one of 1980; that is, the integration into organisational network connections, with strong network centrality, positions across multiple sectors and affiliations, and top organisational roles reinforced by symbolic resources. In 2015, the axis rather corresponded to an opposition between non-Swiss university professors in engineering/natural sciences with a working-class background and sitting on the board of industrial companies, on the one hand, to well-established Swiss elites from the financial sector, with large network connections, symbolic resources and an upper or upper-middle-class social origin (sometimes with a father who was an elite member and company owner himself), on the other.

Several observations can be made on the evolution of the Swiss field of power over 105 years. First, economic capital and social and organisational capital constituted the main forms of power over time. They were rarely accumulated together (at least not on the first axis). Second, inherited wealth mostly structured economic capital in 1910 and 1937. Since the 1950s, with the increasing importance of company executives and managerial capitalism (Jones, 2005), large company owners and top managers allied themselves to defend the interests of the corporate class (Useem, 1982). Established and delegated forms of economic capital often accumulated together afterwards. Third, cultural and symbolic capital were not insignificant, but lacked institutional context for their consecration like in some other European countries. Consequently, they often consolidated other forms of capital (economic, social and organisational), rather than producing power on their own. Fourth, we identified three historical moments. At the beginning, at times of monstrous wealth accumulation, wealth opposed social and organisational capital, as rich capitalists did not need network embeddedness while elites with delegated power from other sectors (especially politics) relied the most on organisational connections. Then, since the 1940s, social-democratic powers, workers’ unions and civil society movements became increasingly present in the neo-corporatist state system, and the effective agents of the field of power resembled Mills’ (1956) coordinated and integrated power elite the most. In this context, established and delegated economic capital still corresponded to the main societal power, while network connections constituted the second one. This time, in the quadrant where the volume of both forms of assets was the highest, a group of dominant ‘hyper-agents’ (Maclean et al., 2017) could single-handedly accumulate society’s main forms of power. Finally, in the most recent period, economic and organisational network powers once again opposed one another, and no individual was structurally capable of accumulating both at the same time any more. This process was concomitant with a documented decrease in solidarity and integration in business, as the largest companies and the billionaires owning them had become increasingly effective in defending their own narrow interests and, much like before the Second World War, did not need network embeddedness any more (Mach et al., 2021; Mizruchi, 2013; Moran, 2008). This marked a return to past logics, as the current main object of struggle in the field of power resembles the one before 1945, when wealth and economic inequality was as high as in the present (Piketty, 2014).

## Conclusions and Discussion: The Field of Power, Social Change and the Weight of the Past

We investigated the most important forms of capital in the Swiss field of power by focusing on the structure of a small group of effective agents during a period of 105 years. We showed that economic and organisational network powers constituted the two most dominant resources throughout history. While before 1945 wealth opposed organisational network connections, between the 1950s and the 1980s economic capital and social and organisational capital could be accumulated together. However, in the recent period, economic capital opposed organisational network connections once again. Wealthy capitalists and their representatives are now as disconnected from organisational and state powers as they were in the pre-war era. While the height of social democracy, neo-corporatism and the *Trente Glorieuses* seems long gone, and as wealth accumulation has increased among a fraction of the elite, we witness a return to former logics. In that sense, the present carries the weight of history and reproduces past social structures.

We provided a comprehensive picture of forms of capital across history. To our knowledge, our research is the first to focus on the evolution of the field of power on such a long time. Our results highlighted the specificities of the Swiss case. In this context, certain forms of capital mattered as much as in other countries, such as economic and social capital or elite seniority, but are subject to different configurations. While in Norway (Denord et al., 2011; Hjellbrekke et al., 2007) and France (Denord et al., 2018) the integration into the economic order constitutes the field’s main form of distinction, in contemporary Switzerland the integration (and seniority) into the private economy also implies the non-integration into the most powerful individual-organisation networks. However, in the 1950s and the 1980s, the field’s main opposition resembled much more contemporary French and Norwegian cases. In Denmark (Lunding et al., 2021), while the field’s main principle corresponds to the overall volume of capital, in contemporary Switzerland several forms of capital are not accumulated together along the first axes. Moreover, some resources that are influential in other countries do not have much impact in the Swiss case. While cultural capital has a strong importance for French (Bourdieu, 1996a) and British (Friedman and Reeves, 2020; Reeves and Friedman, 2024; Reeves et al., 2017; Rossier et al., 2024) elites, linked to the prevalence of elite schools and universities/*Grandes écoles*, in Switzerland culture is less of a distinguishing factor. Similarly, while the French and British states are prone to award (quasi)nobility titles (e.g. the *Légion d’honneur*) to the elites with the highest reputation, the Swiss state is not in a position to do so. Those differences and similarities between national elites have to be kept in mind when undertaking comparative research between different elite contexts (Friedman et al., 2024; Savage and Hjellbrekke, 2021).

Our research has important implications for the study of social change. While a little more than a century is in any case not comparable to the multi-secular studies undertaken by the French *Annales* (see Braudel, 1972), Elias (1969), Zinn (1980) or Mann (1986), our work owes a great debt to such historical research. We also acknowledge the legacy of social change theorists who were able to make sense of rapid evolutions over the course of the 20th century (Bauman, 2000; Beck, 1992). Nevertheless, we did not embrace concepts such as ‘risk society’ or ‘modernity’ implying the decline in importance of social structures, social classes or the elites. We were rather inspired by the ambition of those scholars to describe historical changes over a period of more than one generation, and used Bourdieusian concepts such as field, capital and habitus to do so (Gorski, 2013). Following Savage’s (2023) call for more studies carefully examining long-term social trends and historical shifts to revive ‘big picture sociology’, we showed the impact and permanence of past power structures in contemporary society. While we included a fairly small number of individuals in our analysis, they constituted the most central and powerful individuals among a much larger group of elites. The number of used variables, which is relatively high for a prosopographical field analysis, corresponded to the main forms of power in the field. Through this research strategy, we enlarged the scope of our analysis and contributed to the study of broad social and historical trends. We analysed social change by focusing on the dominant individuals within the field of power, who have a disproportionate influence over society. By a society-wide diffusion process that is not unrelated to the forms of ‘imitation’ and ‘inspiration’ of the ‘lower classes’ formulated by Maurice Halbwachs in the opening quote from this article, the outcome of the definition struggles among this small group of tens to hundreds of individuals becomes the main period-specific stake or *Zeitgeist* (Krause, 2019). This process is obviously not unilateral, as evolutions in the composition of the field of power result from society-wide dynamics of distribution of capital. Nevertheless, highlighting changes in the field of power helped us to make sense of historical societal dynamics.

Our study is not without limitations. Our positional and formal approach on elite networks to identify effective agents implied less focus on informal forms of power among elites. Moreover, our historical perspective, given the number of selected organisations, had to concentrate on a small number of years, which led us to exclude a more fine-grained year-by-year analysis. Finally, our work could have been supplemented by a more qualitative analysis, such as interviews, to understand how power is effectively exerted within this group. Despite those limitations, we provided evidence of past structures influencing present elite constellations on at least three levels. First, at the individual level, elites have personal histories. Their upbringings, schooling, higher education and occupational trajectories (Bühlmann et al., 2024; Ellersgaard et al., 2019) allowed their selection into the elite and paved the way for their current position and social surface in elite fields (Bühlmann, 2010; Toft, 2018, 2019). Second, forms of capital are transmitted from one generation to the other. Elites from the upper class often have acquired class-specific forms of habitus thanks to their parents, allowing them to navigate within elite circles (Hartmann, 2000). The reproduction of parental occupation in the economic fractions of the upper class is particularly critical in securing economic capital. Third, contemporary societies carry the weight of the past through mechanisms that go beyond personal or intergenerational accumulation. In the Swiss field of power, economic and organisational network powers endured over time. In the frame of a return to past inequality logics, the present structural configurations where *wealth does not need network embeddedness any more* resembles the ones from over a century ago, when European imperial powers were ruling over the world and Switzerland was on the verge of becoming one of the main tax havens for the empires (Guex, 2022; Ogle, 2017). Capital accumulation is part of a three-level (*biographical*, *generational* and *structural*) temporal dynamic and helps us to make sense of broad historical processes of social change. While our study focused less on the biographical level, we had several indicators of the effective agents’ education and occupational career (e.g. if they had stayed abroad before entering the elite). Further study needs to be made to tackle the relationship between elite biographies and the two other levels thanks to the use of longitudinal methods.

## Supplemental Material

sj-docx-1-soc-10.1177_00380385251322061 – Supplemental material for Forms of Capital, Social Change and the Weight of the Past: The Effective Agents of the Swiss Field of Power 1910–2015 Supplemental material, sj-docx-1-soc-10.1177_00380385251322061 for Forms of Capital, Social Change and the Weight of the Past: The Effective Agents of the Swiss Field of Power 1910–2015 by Thierry Rossier and Jacob Aagaard Lunding in Sociology

## Notes

1. We focus on those six periods with, each time, a year, which is assumed to be representative of a timeframe of about 20 years. We are conscious that such an approach does not come without limitations since we are not able to focus on elites’ profiles on a year-to-year basis. Nevertheless, as elite spaces do not change quickly in short periods of time (Ellersgaard and Larsen, 2023), such an approach appeared to us as satisfying enough to grasp century-long changes among the Swiss elites.

2. See the Swiss Elite Database’s website: https://www2.unil.ch/elitessuisses.

3. In previous research we also included 2010 to focus on the global financial crisis. However, this year was not included, since the core had a very distinct composition that impacted the MCA’s structure.

4. Online Appendix 1 gives the organisational composition and properties of the one-mode networks and the cores.

5. This group has been extremely homogeneous in terms of gender. Until 1957, there were no women. In 1980, they were only 4%, which is a too low proportion to include gender in the MCA. They were finally 15% in 2000 and 14% in 2015. All in all, elite network cores are usually mainly composed of (white) men (Rossier and Ellersgaard, 2023).

6. Online Appendix 2 provides more details on the variables.

7. Online Appendix 3 provides inertia rate tables, modality and variable contribution tables and spatialisation of active and supplementary modalities. The number of axes necessary to explain 80% of the adjusted variance rates varies from five to six. Since the first two axes (and especially the first axis) contribute to a very strong share of the overall variance, we decided to mainly focus on those two axes. The appendix also provides information on the third axis.

8. We projected top company ownership as a supplementary characteristic. It went in the same direction as the other economic variables. Its coordinates were the following. 1957: Yes (n = 28; x = 1.7; y = 0.2), No (n = 183; x = −0.3; y = −0.0). 1980: Yes (n = 6; x = 2.2; y = −0.6), No (n = 191; x = −0.1; y = 0.0). The eta-squared correlation coefficient between the axes and the variable was the following. 1957: η² axis 1 = 44%; η² axis 2 = 0%. 1980: η² axis 1 = 15%; η² axis 2 = 0%.
