# Supplementary material for: Forms of Capital, Social Change and the Weight of the Past: The Effective Agents of the Swiss Field of Power 1910–2015
Source: Sociology. 2025 Mar 13;59(4):761–81. doi: 10.1177/00380385251322061 (PMC12306905; doi:10.1177/00380385251322061)
Supplement: sj-docx-1-soc-10.1177_00380385251322061 – Supplemental material for Forms of Capital, Social Change and the Weight of the Past: The Effective Agents of the Swiss Field of Power 1910–2015 [file sj-docx-1-soc-10.1177_00380385251322061.docx]

# Online Appendix 1: Summary of the Swiss elite network

*Table A: Types of affiliations in the two-mode Swiss elite network (absolute numbers)*

| **Elites** | **Type of organisations** | **1910** | **1937** | **1957** | **1980** | **2000** | **2015** |
| --- | --- | --- | --- | --- | --- | --- | --- |
| Academia | Associations and academic organisations | 4 | 4 | 10 | 10 | 11 | 11 |
|  | Universities | 8 | 9 | 9 | 10 | 12 | 12 |
| Administration | Supreme court | 1 | 1 | 1 | 1 | 1 | 1 |
|  | Central bank | 1 | 1 | 1 | 1 | 1 | 1 |
|  | Federal departments | 8 | 8 | 8 | 8 | 8 | 8 |
| Expertise | Expert committees | 58 | 72 | 214 | 298 | 183 | 143 |
| Business | Top companies | 112 | 110 | 108 | 107 | 112 | 109 |
|  | Main business associations | 5 | 5 | 5 | 5 | 5 | 5 |
| Unions | Main unions | 2 | 2 | 2 | 2 | 2 | 2 |
| Politics | Federal government | 1 | 1 | 1 | 1 | 1 | 1 |
|  | Federal parliament | 2 | 2 | 2 | 2 | 2 | 2 |
|  | Cantonal governments | 25 | 25 | 25 | 26 | 26 | 26 |
|  | Largest city governments | 4 | 4 | 4 | 4 | 4 | 4 |
|  | Main political parties | 2 | 4 | 4 | 4 | 5 | 7 |
| Interest associations | Most influential associations and organisations | 14 | 20 | 20 | 23 | 23 | 9 |
| Military | The generals | 1 | 1 | 1 | 1 | 1 | 1 |
| **Total** |  | **248** | **269** | **415** | **503** | **397** | **342** |

To build the two-mode elite network, individuals were divided into eight main categories according to the sector of the institutions they were connected to (one individual can be affiliated to more than one sector or type of organisation), before being projected into a one-mode elite network with the affiliations making the ties between individuals. For *academia* we included the executive board members of the main scientific associations and academic organisations but also university presidents (rectors) and department deans of all cantonal universities and federal technical schools. For *public administration* we selected the members of the supreme court, the directory members of the Swiss National Bank (Swiss central bank) and, in the seven federal departments and the federal chancellery, we included the seven elected ministers (federal councillors), the chancellor and vice chancellors, the general secretaries of each department and the directors of each federal office. For *expertise* we included all corporatist expert committee (extra-parliamentary commissions) members for the federal administration. For *business* we included the CEOs, delegates of the board and other (non-executive) board members of the largest *.ca* 110 Swiss companies (according to turnover, number of employees, assets and market capitalisation^[[1]](#footnote-1)^) and the executive committee members of the five most important business associations. For the *union* sector, the two most influential union federations were included. For *politics*, we selected the seven members of the federal government (Federal Council), the members of the Senate (Council of State) and the House of Representatives (National Council) of the federal parliament, the members of all the cantonal governments, the members of the governments of the largest cities (Zurich, Geneva, Bern and Lausanne; the governments of the city and the canton of Basel are the same institution) and the executive committee members of the main political parties. For the *interest associations* (other than the main business associations and unions) we included the board members of other important associations, foundations, societies, non-governmental organisations and think tanks. Finally, for the *military*, we retained the highest-ranking generals (*Korpskommandanten*).

*Table B: Properties of the one-mode Swiss elite network and of the elite core*

|  | **1910** | **1937** | **1957** | **1980** | **2000** | **2015** |
| --- | --- | --- | --- | --- | --- | --- |
| **Elite individuals** |  | | | | | |
| Total | 1584 | 1877 | 3110 | 4475 | 3738 | 3651 |
| Elite core (k-shells) | 75 | 103 | 211 | 197 | 96 | 49 |
| Elite core (k-shells) % | 4.7% | 5.5% | 6.8% | 4.4% | 2.6% | 1.3% |
| **Edges** |  | | | | | |
| Total | 26 818 | 35 848 | 61 274 | 77 941 | 60 925 | 56 239 |
| Elite core (k-shells) | 601 | 900 | 3991 | 2977 | 772 | 243 |
| Elite core (k-shells) % | 2.2% | 2.5% | 6.5% | 3.8% | 1.3% | 0.4% |
| **Graph density** |  | | | | | |
| Entire elite network | 0.021 | 0.020 | 0.013 | 0.008 | 0.009 | 0.008 |
| Elite core (k-shells) | 0.217 | 0.171 | 0.180 | 0.154 | 0.169 | 0.207 |
| **Average path length** |  | | | | | |
| Whole elite network | 3.297 | 3.188 | 3.163 | 3.469 | 3.818 | 4.307 |
| Elite core (k-shells) | 1.967 | 2.048 | 2.021 | 2.091 | 2.231 | 1.832 |
| **Transitivity** |  | | | | | |
| Entire elite network | 0.863 | 0.871 | 0.718 | 0.738 | 0.877 | 0.925 |
| Elite core (k-shells) | 0.546 | 0.461 | 0.460 | 0.434 | 0.546 | 0.633 |
| **K-score** |  | | | | | |
| Mean k-score | 33 | 56 | 93 | 89 | 41 | 25 |
| Elite core (k-shells) % reached with mean score | 43.6% | 53.9% | 44.1% | 45.0% | 43.1% | 50.1% |

# Online Appendix 2: Variables to measure the Field of Power

*Table C: Blocks of variables, variables and modalities in the specific MCAs*

| **Dimensions** | **Variables** | **Indicators** | **1910** | **1937** | **1957** | **1980** | **2000** | **2015** | **Comments** |
| --- | --- | --- | --- | --- | --- | --- | --- | --- | --- |
| Inherited capital and seniority | Gender | Woman |  |  |  |  |  |  | No women in the group before 1980, too small numbers before 2000. |
|  |  | Man |  |  |  |  |  |  |  |
|  | Language | German |  |  |  |  |  |  | Other (Non-Swiss): too small numbers. |
|  |  | French/Italian/Romansch |  |  |  |  |  |  |  |
|  |  | Other (Non-Swiss) |  |  |  |  | p | p |  |
|  | Citizenship | Swiss |  |  |  |  |  |  | Non-Swiss (1937-2000): too small numbers. |
|  |  | Non-Swiss |  |  |  |  |  |  |  |
|  | Age | 30-49 |  |  |  |  |  |  |  |
|  |  | 50-59 |  |  |  |  |  |  |  |
|  |  | 60-69 (60+) |  |  |  |  |  |  |  |
|  |  | 70+ |  |  |  |  |  |  |  |
|  |  | Missing |  | p | p | p | p |  |  |
|  | Belongs to former agents period | Yes |  |  |  |  |  |  | 1910: no information; 1957: too associated with class/others; 2015: too small numbers. |
|  |  | No |  |  |  |  |  |  |  |
| Social and family back-ground | Father's class | Upper class |  |  |  |  |  |  | Working class (and lower middle class in 1910 ): too associated with father's occupation. |
|  |  | Upper middle class |  |  |  |  |  |  |  |
|  |  | Lower middle class | p |  |  |  |  |  |  |
|  |  | Working class | p | p | p | p | p |  |  |
|  |  | Missing | p | p | p | p | p | p |  |
|  | Father elite | Yes |  |  |  |  |  |  |  |
|  |  | No |  |  |  |  |  |  |  |
|  | Father's occupation | Company owner |  |  |  |  |  |  |  |
|  |  | Executive |  |  |  |  |  |  |  |
|  |  | Liberal profession |  |  |  |  |  |  |  |
|  |  | Executive/Liberal profession |  |  |  |  |  |  |  |
|  |  | Intellectual occupation |  |  |  |  |  |  |  |
|  |  | Small independent |  |  |  |  |  |  |  |
|  |  | Farmer |  |  |  |  |  |  |  |
|  |  | Intermediary occupation |  |  |  |  |  |  |  |
|  |  | Worker |  |  |  |  |  |  |  |
|  |  | Other occupation |  |  |  |  |  |  |  |
|  |  | Missing | p | p | p | p | p | p |  |
|  | Father-in-law elite | Yes |  |  |  |  |  |  | 2000-2015: too small numbers. |
|  |  | No |  |  |  |  |  |  |  |
|  | Citizenship of the spouse | Swiss |  |  |  |  |  |  | Single: too small numbers. |
|  |  | Non-Swiss |  |  |  |  |  |  |  |
|  |  | Single | p | p |  | p |  | p |  |
|  |  | Missing | p | p | p | p | p | p |  |
|  | Family ties in the agents (in same period) | Yes |  |  |  |  |  |  | 1957: too associated with class/others; 1980-2015: too small numbers. |
|  |  | No |  |  |  |  |  |  |  |
| Cultural capital | Highest diploma | Doctorate |  |  |  |  |  |  |  |
|  |  | University licence |  |  |  |  |  |  |  |
|  |  | Vocational |  |  |  |  |  |  |  |
|  |  | Missing | p | p | p | p |  |  |  |
|  | Discipline of university diploma | Law |  |  |  |  |  |  | No discipline: same as vocational education and no university; Medicine: too small numbers; Social Sciences & Humanities (1957): too small numbers. |
|  |  | Technical/Natural Sciences |  |  |  |  |  |  |  |
|  |  | Economics/Business |  |  |  |  |  |  |  |
|  |  | Social Sciences/Humanities |  |  | p |  |  |  |  |
|  |  | Medicine |  |  | p | p | p | p |  |
|  |  | No | p | p | p | p | p | p |  |
|  |  | Missing | p | p | p | p |  |  |  |
|  | University of diploma | Top |  |  |  |  |  |  | No university: same as vocational education and no university; Other country (1980): too small numbers; Top = ETHZ, Zurich, Geneva, Basel; Mid-range = Lausanne, Bern; Bottom = EPFL, Fribourg, Neuchâtel, St.Gallen, other Swiss university institute. |
|  |  | Mid-range |  |  |  |  |  |  |  |
|  |  | Bottom |  |  |  |  |  |  |  |
|  |  | Mid-range/Bottom |  |  |  |  |  |  |  |
|  |  | Other country |  |  |  | p |  |  |  |
|  |  | No | p | p | p | p | p | p |  |
|  |  | Missing | p |  | p | p |  |  |  |
|  | MBA | Yes |  |  |  |  |  |  | MBAs: only significant for the 2000 and 2015 periods. |
|  |  | No |  |  |  |  |  |  |  |
|  | Professor | Yes |  |  |  |  |  |  | 1910: too small numbers. |
|  |  | No |  |  |  |  |  |  |  |
| Economic capital | Economic sub-sector | Banking and finance |  |  |  |  |  |  | No economic subsector (1910): too small numbers. |
|  |  | Industry |  |  |  |  |  |  |  |
|  |  | Commerce |  |  |  |  |  |  |  |
|  |  | Industry/Commerce |  |  |  |  |  |  |  |
|  |  | No economic subsector | p |  |  |  |  |  |  |
|  | Company manager | Yes |  |  |  |  |  |  |  |
|  |  | No |  |  |  |  |  |  |  |
|  | Company chair | Yes |  |  |  |  |  |  |  |
|  |  | No |  |  |  |  |  |  |  |
|  | Company board | Yes |  |  |  |  |  |  | 1910: too small numbers. |
|  |  | No |  |  |  |  |  |  |  |
|  | Top 110 company owner | Yes |  |  |  |  |  |  | 1957: too associated with other economic variables; 1980: too small numbers. |
|  |  | No |  |  |  |  |  |  |  |
| Social capital | Geography | City |  |  |  |  |  |  |  |
|  |  | Countryside |  |  |  |  |  |  |  |
|  | Reach=2 in the effective agents | Low |  |  |  |  |  |  |  |
|  |  | Medium |  |  |  |  |  |  |  |
|  |  | High |  |  |  |  |  |  |  |
|  | Betweenness in main component | Low |  |  |  |  |  |  |  |
|  |  | Medium |  |  |  |  |  |  |  |
|  |  | High |  |  |  |  |  |  |  |
|  | Parliament | Yes |  |  |  |  |  |  | 2015: too small numbers. |
|  |  | No |  |  |  |  |  |  |  |
|  | Expert committee | Yes |  |  |  |  |  |  |  |
|  |  | No |  |  |  |  |  |  |  |
|  | Academic organisation | Yes |  |  |  |  |  |  | 1910, 1937 and 2000: too small numbers. |
|  |  | No |  |  |  |  |  |  |  |
|  | Influential association | Yes |  |  |  |  |  |  | 1980: too small numbers. |
|  |  | No |  |  |  |  |  |  |  |
|  | Number of sectors | 1 |  |  |  |  |  |  |  |
|  |  | 2 |  |  |  |  |  |  |  |
|  |  | 3 (3+) |  |  |  |  |  |  |  |
|  |  | 4+ |  |  |  |  |  |  |  |
| Symbolic capital | Prestigious organisation | Yes |  |  |  |  |  |  |  |
|  |  | No |  |  |  |  |  |  |  |
|  | Prominent last name | Yes |  |  |  |  |  |  |  |
|  |  | No |  |  |  |  |  |  |  |
|  | Dr. honoris causa | Yes |  |  |  |  |  |  |  |
|  |  | No |  |  |  |  |  |  |  |
|  | Media presence | None/None or very low |  |  |  |  |  |  |  |
|  |  | Low |  |  |  |  |  |  |  |
|  |  | Medium |  |  |  |  |  |  |  |
|  |  | High |  |  |  |  |  |  |  |
| Organisa-tional capital | Stay abroad | Yes |  |  |  |  |  |  |  |
|  |  | No |  |  |  |  |  |  |  |
|  |  | Missing | p | p | p | p | p |  |  |
|  | Number of affiliations | 2 |  |  |  |  |  |  |  |
|  |  | 3 |  |  |  |  |  |  |  |
|  |  | 4 |  |  |  |  |  |  |  |
|  |  | 5 (5+) |  |  |  |  |  |  |  |
|  |  | 6+ |  |  |  |  |  |  |  |
|  | Non-company executive | Yes |  |  |  |  |  |  |  |
|  |  | No |  |  |  |  |  |  |  |
|  | Non-company chair | Yes |  |  |  |  |  |  |  |
|  |  | No |  |  |  |  |  |  |  |
|  | Military rank | Colonel/General |  |  |  |  |  |  |  |
|  |  | Major/Lieutenant colonel |  |  |  |  |  |  |  |
|  |  | Lieutenant/Captain |  |  |  |  |  |  |  |
|  |  | No |  |  |  |  |  |  |  |
|  |  | Missing |  |  |  |  |  | p |  |
| **Supplementary variable** | | | | | | | | | |
| Sector | Main sector | Companies |  |  |  |  |  |  |  |
|  |  | Business associations |  |  |  |  |  |  |  |
|  |  | Business |  |  |  |  |  |  |  |
|  |  | Politics |  |  |  |  |  |  |  |
|  |  | Unions |  |  |  |  |  |  |  |
|  |  | Academia |  |  |  |  |  |  |  |
|  |  | Unions and academia |  |  |  |  |  |  |  |
|  |  | Administration |  |  |  |  |  |  |  |

*Notes: Black cell: the variable was not used at the year; Grey cell: the modality was not existing at the year; p: the modality was projected as passive. Missing values were projected as passive, as well as values forming less than 5% of the total distribution and modalities too associated with others (in that case, for a binary variable, the variable was not used).*

*Blocks of variables and indicators*

***1 Inherited capital and seniority***

This bloc of variables relates to inherited characteristics as well as to the time spent in the field of power. It does not correspond to any particular form of capital, but rather work in conjunction with other resources. The used indicators are the following:

- **Gender** is measured by the person’s *sex*. Women were not present in the core/effective agents until very recently.
- **Language** is measured by the *language spoken during the primary socialisation* (mediated by the language spoken at the place of birth, of early schooling, of studies or of early jobs; in the rare cases where a doubt subsisted, we looked at the first and last name of the person). In Switzerland, the (Swiss) German-speaking population composes the large majority and German usually is the dominant language in national institutions. Therefore, we recoded the languages from the three linguistic minorities (French, Italian, Romansch) altogether.
- **Citizenship** is measured by the *nationality at birth*. Switzerland is one of the countries with the highest rate of foreigners among its elite. They were more present at the beginning and during the most recent period.
- **Age** is measured by the person’s *biological age*.
- **Belonging to the former effective agents’ period** is measured by whether or not the *person was present in the previous period of the elite core*. This indicator of elite seniority is not available for the members of the 1910 period, as we did not have information on a former core.

***2 Social and family background***

This bloc of variables relates to inherited resources and resources acquired by marriage. Some variables do not reflect a particular form of capital, but rather relate to an overall volume of capital (class of origins, father and father-in-law member of the elites, family ties in the effective agents). However, with the occupation of the father, we can identify a form of inherited economic capital (when the father was a company owner or to some extent when he was an executive or a professional) and of inherited cultural capital (father had an intellectual occupation). The indicators are the following:

- **Class background** is measured by the *occupation of the father* (as often no information could be found on the occupation of the mother). We used the following class scheme: *Upper class*: father was a large company owner, occupied another prominent (elite) position in Swiss politics or in business, came from a professional or intellectual occupational background (e.g. lawyer, medical doctor, university professor, artist) with family ties to wealthy or old patrician families, or occupied an executive position in an important Swiss organisation; *Upper middle class*: owner, tradesperson or executive in mid-size organisations, public figure of regional importance, farmer from medium to large enterprises, liberal professions (lawyer, engineer, doctor), intellectual occupations (university professor, priest, artist), regional civil servant; *Lower middle class*: small tradesperson or other small entrepreneur, craftsperson, farmer; *Working class*: skilled or unskilled worker or employee. Therefore, this scheme tries not only to take in consideration the occupation of the father, but also the size of the organisation, the prestige and level of the position, the accumulated wealth or the family ties with old and wealthy dynasties.
- **Father member of the elite** is measured by whether or not the person’s *father had been a member of the Swiss elites* at some point according to the same definition as described in Appendix 1.
- **Parental occupation** is measured by the *occupation of the person’s father*. This indicator only relies on the occupational characteristics, contrary to the class background indicator.
- **Father-in-law member of the elite** is measured by whether or not the person’s *father-in-law had been a member of the Swiss elites* at some point according to the same definition as for the father.
- **Citizenship of the spouse** is measured by the *nationality of the person’s spouse*. This indicator corresponds to a proxy for the endowment in cosmopolitan capital (Bühlmann et al., 2013) acquired through marriage.
- **Family ties in the effective agents (in the same period)** is measured by whether or not the person is *related by family links within the elite core*. Family ties in the core include: parents, siblings, children, first and more distant cousins, uncles/aunts, nephews/nieces, parents-in-law, siblings-in-law, children-in-law and other family-in-law.

***3 Cultural capital***

This group of variables relates to formalised cultural capital. As mentioned in the article, there are no elite schools and universities in Switzerland and as a result the entrance to top higher education institutions depends less on the belonging to the upper-class and its class-specific habitus ownership. The indicators are the following:

- **Highest diploma** is measured by the *highest educational level*.
- **Discipline of university diploma** is measured by the *field of the highest university diploma, if any*.
- **University of diploma** is measured by the *institution of the highest university diploma, if any*. For the Swiss universities, we proceed to a rough ranking of these institutions based upon their size, the size of their home city (Zurich, Geneva and Basel being the three largest cities) and their seniority (as some of these universities were created in the 19^th^ century, or even before for Basel). While proceeding to this ranking, we kept in mind that no university in Switzerland is considered an elite institution and that this ranking is not completely straightforward, but rather helped us to divide those institutions by size and scope. On the *top*, we classified the universities of Zurich, Geneva, Basel and the ETH Zurich. As *mid-range* universities, we classified the universities of Lausanne and Bern. On the *bottom* we classified smaller and/or specialised universities from smaller towns: the EPF Lausanne, the universities of Fribourg, Neuchâtel and St. Gallen, as well as other university institutes. While the EPFL is currently considered as a top university worldwide, ranking high in global university rankings, before 2000 it only was a provincial engineering school. The University of Fribourg is less specialised and larger than the others from the same category, but it has always been the university of the country’s dominated rural Catholic population. Finally, the University of St. Gallen has been recently known to be the alma mater of many business elites, but it also is a small university with a specialisation in management.
- **MBA/executive program** is measured by whether or not the person has received *an executive diploma in management*. Masters in business administration were not really relevant in the business sector before the 1990s.
- **University professor** is measured by whether or not the person has occupied a *professor position at a university*, in Switzerland or in another country. A professor position relates to one of the highest forms of cultural capital in the social space.

***4 Economic capital***

As economic capital is particularly critical for distinction processes among the Swiss elites, we included this block of variables. Among those indicators, we differentiate between **established economic capital,** which is measured by being a large Swiss company owner and is a good proxy for (often inherited) personal wealth, and **delegated economic capital**, measured by positions as company manager, chair of the board or other board member of a large Swiss company. The indicators are the following:

- **Economic subsector** is measured by whether or not the person is sitting on *the board of one of the 110 largest Swiss companies in the banking and financial sector, the industrial sector or the commercial sector*. In the case of multiple company affiliations, the sector of the company with a CEO, chair or board delegate position prevails over the other board member positions. In the case of affiliations to more than one sector, the banking and finance sector prevails over the industrial one, and the industrial sector prevails over the commercial one.
- **Company manager** is measured by whether or not the person occupies a *managerial position in one of the 110 largest Swiss companies*.
- **Company chair** is measured by whether or not the person occupies a *chair of the board position in one of the 110 largest Swiss companies*.
- **Company board** is measured by whether or not the person occupies *another position in the non-executive board in one of the 110 largest Swiss companies*.
- **Top 110 company owner** is measured by the fact that the person is *owner of one of the 110 largest Swiss companies*. As we were not able to retrieve rich lists for the entire 1910-2015 period, this variable constitutes the most accurate proxy for personal wealth. Since 1989, the magazine *Bilanz* published annually the list of the 100, then 300, richest people and family dynasties in Switzerland. When comparing our indicator with those lists for the years 1980, 2000 and 2015, we saw that those lists overlapped almost completely with it, proving the relevance of our indicator. Since this indicator was too associated with others in 1957 and formed less than 5% of the total distribution in 1980, we were not able to project it as an active variable but projected it later as a supplementary property.

***5 Social capital***

This bloc of variables related to social capital corresponds to geographical features, centrality in the affiliation network, affiliation to organisations where elites from several sectors meet and multipositionality across sectors. The indicators are the following:

- **Elite geography** is measured by whether the person’s *main organisation of affiliation is located in a city or in the countryside*. The six Swiss largest cities (Zurich, Geneva, Basel, Bern, Lausanne and Winterthur) are qualified as cities and the rest of the country corresponds to the countryside. It is a measure of social capital since in cities the concentration of elites is higher and so are the odds to meet other members of the elites.
- **Reach=2 in the effective agents** is measured by the *number of people reached within two steps in the one-mode elite core network at each year*. We divided each time this variable into three categories of similar size.
- **Betweenness in the main component of the Swiss elite network** is measured by the number of times the person falls on the *shortest path between two other people in the one-mode Swiss elite network at each year*. We divided each time this variable into three categories of similar size.
- **Parliament** is measured by whether or not the person is *sitting in one of the two chambers of the Swiss federal parliament*.
- **Expert committee** is measured by whether or not the person is *sitting in a state expert committee for the federal administration (extra-parliamentary commissions)*.
- **Academic organisation** is measured by whether or not the person is *sitting in an academic society or a scientific association*.
- **Influential association** is measured by whether or not the person is *sitting in an influential association, foundation, society or think tank*.
- **Number of sectors**, which is a proxy for multipositionality, is measured by *the total number of sectors covered at the time through elite affiliations over a total of nine key sectors* (companies, business associations, unions, politics, administration, academia, expert committees, other types of interest associations and the military – rank between lieutenant and general).

***6 Symbolic capital***

This sixth block of variables relates to resources linked to processes of reputation, recognition and prestige. As mentioned in the paper, there is no award, medals or titles granted by the state or a monarch in Switzerland, therefore symbolic capital must rather be found through other forms of recognition. We used the following indicators:

- **Prestigious organisations** is measured by whether or not the person is *sitting on one of the most prestigious organisations in the elite network*. In that case, in link with the idea of capital accumulation, we assess prestige in a historical perspective by taking the list of the organisations which are present in the network of the elite core without discontinuation during the whole period (i.e. for each of the six years). This calculation ended up on a very restrained list of eight organisations in total: the senate/upper chamber of the parliament (state council); the bank council of the Swiss National Bank (the central bank); two business associations: the Swiss Union of Commerce and Industry (later EconomieSuisse) and the Swiss Bankers Association; and four companies: Credit Suisse (bought by UBS in 2023), Nestlé, Bâloise Holding and the Swiss Bank Corporation (which becomes UBS in 1998 after a merger with Union Bank of Switzerland). Sitting on at least one of those eight organisations in the elite network provides effective agents with a large amount of symbolic capital.
- **Prominent last name** is measured by whether or not the individual has a *recognised family name*. Like for prestigious organisations, we conceptualised the symbolic power of names in a historical perspective. In the elites and the upper classes, patronyms are markers of belonging to the group and of inherited capital from the highest circles from society. Especially, they are particularly efficient at the local level where powerful dynasties rule municipalities. Thanks to data gathered in the frame of the “Local Power Structures and Transnational Connections. New Perspectives on Elites in Switzerland, 1890-2020” SNSF Sinergia project (grant number: 183534), we could focus on family names from the three largest cities of Zurich, Basel and Geneva, where the most powerful old families produce influential elites at the national level and constitute a form of local nobility (Benz et al., 2024; Mach et al., 2024). The names of the elites from the three cities have been collected in the frame of the project. Individual selected were the following: the cities’ largest company executive and non-executive board members, business associations (chambers of commerce) leaders, MPs and members of the government of the three cantons and three cities, members of fine arts societies and university professors at the historical benchmark years of 1890, 1910, 1937, 1957, 1980, 2000 and 2020. We then considered a family name (excluding family names the individuals acquired by marriage) to be a prominent one among local elites when at least two people had the same family name among the elites from the same city for at least three years out of the seven in total (in total we obtained a list of 94 patronyms by following this rule). Therefore, we could assume that these people were part of a long-lasting elite dynasty reproducing a family name over several generations. Afterwards, we compared the list with the names of the effective agents. In order not to give too much weight to very common names in the Swiss population, which would have less symbolic power among the elites, even if they belong to powerful dynasties, we removed from our list the 6 most common names in 2021 (knowing that they were quite stable historically): “Müller”, “Meier”, “Schmid”, “Keller”, “Weber” and “Schneider”.
- **Honorary doctorate** is measured by whether or not the person received a *honorary title from a university*. These distinctions, which are awarded based on the “merit” of a person, in academia or outside, are attached to a large amount of symbolic power as universities in Switzerland own a lot of this form of capital. They should not be considered as a form of institutionalised cultural capital, as they are not necessarily awarded to people who own university diplomas. We obtained the list of all doctorates *honoris causa* from the Swiss universities to establish the list of people among the effective agents who obtained such a title.
- **Media coverage** is measured by the *number of mentions the person has had in a large list of Swiss newspapers during the year in question and until ten years before that*. To do so we collected automatically these mentions thanks to the <https://www.e-newspaperarchives.ch/> website (last accessed September 28^th^ 2023), which currently contains a collection of 180 digitalised Swiss newspapers, among which many were already published in the 19^th^ century, and constitutes the country’s largest historical collection of digitalised press. As the majority of the press in Switzerland is either published in German or French, we only focused on the articles published in those two languages, discarding occurrences in Italian and Romansch. To identify the person, we searched for the following terms “first name last name” (with no more than one word between them, in case of a middle name) *and* “affiliation(s)” (if multiple affiliations: “affiliation 1 *or* affiliation 2 *or* affiliation 3 etc.”). We used the main organisations the person was affiliated at the time, using all their possible names (in French and German). We tried multiple solutions to identify mentions of the individuals before opting for this choice. The mention of only the first and last name, including or not the prefixes (“Dr.”, “Professor”) if any, without the affiliations, led to too many homonyms. So was the mentions of only the last name and the affiliations. Eventually, first and last name *and* affiliation was the best solution, as while increasing the accuracy of the search by decreasing the number of false positives, an elite individual is anyway rarely mentioned in the media without also mentioning their organisational affiliation providing symbolic credit to them. We divided each time this variable into four categories of similar size (except in 1910 when a large portion of the effective agents where not mentioned the press).

***7 Organisational capital***

This block of variables corresponds to other types of resources, linked to credentials provided by the connected organisations in affiliation networks, the position in those organisations and other career properties that also form the specific capital of the field of power. The indicators are the following:

- **Stay abroad** is measured by whether or not the person has *stayed outside Switzerland for a substantial period of time (i.e. about at least a year) during their studies or their career before the considered year*. Having stayed abroad is a measure for cosmopolitan capital (Bühlmann et al., 2013).
- **Number of affiliations** is measured by the *number of organisations the person is directly linked to in the two-mode elite network at the year*.
- **Non-company executive** is measured by whether or not the person *occupies an executive position in one organisation in the two-mode elite network outside of companies* (i.e. military general, member of a government, supreme court member, governor of the central bank, executive secretary of a federal department, head of a federal office, executive secretary of a business association or a union, or head of an academic department or of a university).
- **Non-company chair** is measured by whether or not the person *occupies a chair of the board position in one organisation in the two-mode elite network* (i.e. chair of an expert committee, of a political party, of a business association, of a union, of an academic organisation, of a scientific association or of any other kind of association).
- **Military rank** is measured by the person’s *highest officer rank in the Swiss militia army, if any*. This information was partly retrieved from the annual publication of the *Offiziers-Etat/Etat des officiers* digitalised by the federal administration from the period 1893 onwards, with however a thirty-year moratorium.

***A Supplementary variable***

On top of the seven blocs of active variables, as a supplementary variable the **main sector**, which is measured by the person’s *main organisational sector of affiliation*. On top of this variable we also collected information on the person’s *religion*, *region of the main affiliation in Switzerland*, *country of stays abroad* and *political party affiliation*. Those variables were not used as active because of their potential redundancy with other variables, or interpretability.

# Online Appendix 3: The Historical Geometry of Power. Supplementary Material

*Table D: Basic MCA information*

|  | **1910** | **1937** | **1957** | **1980** | **2000** | **2015** |
| --- | --- | --- | --- | --- | --- | --- |
| **Individuals** | 75 | 103 | 211 | 197 | 96 | 49 |
| **Number of active axes** | 21 | 21 | 25 | 25 | 25 | 22 |
| **Axes explaining 80% of inertia** | 5 | 5 | 5 | 6 | 5 | 6 |
| **Active variables** | 32 | 34 | 32 | 32 | 34 | 34 |
| **Active modalities** | 82 | 89 | 89 | 88 | 94 | 92 |
| **Passive modalities** | 13 | 11 | 13 | 14 | 10 | 9 |
| **Share of passive modalities** | 0.05 | 0.03 | 0.04 | 0.05 | 0.06 | 0.04 |

*Table E: Inertia rates of the six MCAs*

| **1910** | | | | | | | | | | |
| --- | --- | --- | --- | --- | --- | --- | --- | --- | --- | --- |
| **Axis** | **1** | **2** | **3** | **4** | **5** | **6** | **7** | **8** | **9** | **10** |
| **Eigenvalue** | 0.18 | 0.11 | 0.10 | 0.09 | 0.08 | 0.07 | 0.07 | 0.06 | 0.06 | 0.06 |
| **Variance (%)** | 11.3 | 7.0 | 6.0 | 5.7 | 5.0 | 4.5 | 4.2 | 3.8 | 3.8 | 3.5 |
| **Adjusted variance (%)** | 46.8 | 14.3 | 9.2 | 7.9 | 5.5 | 4.0 | 3.0 | 2.3 | 2.1 | 1.6 |
| **Cumulated adjusted variance (%)** | 46.8 | 61.1 | 70.3 | 78.2 | 83.7 | 87.7 | 90.7 | 93.0 | 95.1 | 96.7 |
| **1937** | | | | | | | | | | |
| **Axis** | **1** | **2** | **3** | **4** | **5** | **6** | **7** | **8** | **9** | **10** |
| **Eigenvalue** | 0.15 | 0.12 | 0.09 | 0.08 | 0.08 | 0.07 | 0.06 | 0.06 | 0.06 | 0.05 |
| **Variance (%)** | 9.1 | 7.1 | 5.5 | 5.2 | 5.0 | 4.2 | 3.9 | 3.7 | 3.5 | 3.2 |
| **Adjusted variance (%)** | 36.4 | 19.4 | 9.6 | 8.2 | 7.6 | 4.5 | 3.4 | 2.8 | 2.3 | 1.7 |
| **Cumulated adjusted variance (%)** | 36.4 | 55.8 | 65.4 | 73.6 | 81.2 | 85.7 | 89.1 | 91.9 | 94.2 | 95.9 |
| **1957** | | | | | | | | | | |
| **Axis** | **1** | **2** | **3** | **4** | **5** | **6** | **7** | **8** | **9** | **10** |
| **Eigenvalue** | 0.13 | 0.11 | 0.10 | 0.07 | 0.07 | 0.06 | 0.06 | 0.06 | 0.05 | 0.05 |
| **Variance (%)** | 7.1 | 5.9 | 5.5 | 3.9 | 3.6 | 3.4 | 3.2 | 3.1 | 3.0 | 2.7 |
| **Adjusted variance (%)** | 33.0 | 20.3 | 16.5 | 5.8 | 4.7 | 3.6 | 2.8 | 2.5 | 2.2 | 1.5 |
| **Cumulated adjusted variance (%)** | 33.0 | 53.3 | 69.8 | 75.6 | 80.3 | 83.9 | 86.7 | 89.2 | 91.4 | 92.9 |
| **1980** | | | | | | | | | | |
| **Axis** | **1** | **2** | **3** | **4** | **5** | **6** | **7** | **8** | **9** | **10** |
| **Eigenvalue** | 0.15 | 0.10 | 0.09 | 0.07 | 0.07 | 0.06 | 0.06 | 0.06 | 0.05 | 0.05 |
| **Variance (%)** | 8.1 | 5.4 | 4.7 | 4.2 | 3.7 | 3.6 | 3.4 | 3.1 | 3.0 | 2.9 |
| **Adjusted variance (%)** | 43.0 | 15.0 | 10.0 | 6.9 | 4.6 | 4.2 | 3.6 | 2.3 | 2.1 | 1.7 |
| **Cumulated adjusted variance (%)** | 43.0 | 58.0 | 68.0 | 74.9 | 79.5 | 83.7 | 87.3 | 89.6 | 91.7 | 93.4 |
| **2000** | | | | | | | | | | |
| **Axis** | **1** | **2** | **3** | **4** | **5** | **6** | **7** | **8** | **9** | **10** |
| **Eigenvalue** | 0.18 | 0.12 | 0.09 | 0.09 | 0.08 | 0.07 | 0.07 | 0.06 | 0.06 | 0.06 |
| **Variance (%)** | 10.0 | 6.4 | 5.0 | 4.9 | 4.3 | 3.9 | 3.7 | 3.5 | 3.3 | 3.2 |
| **Adjusted variance (%)** | 45.7 | 15.2 | 7.8 | 7.4 | 5.0 | 3.6 | 3.1 | 2.6 | 2.2 | 1.8 |
| **Cumulated adjusted variance (%)** | 45.7 | 60.9 | 68.7 | 76.1 | 81.1 | 84.7 | 87.8 | 90.4 | 92.6 | 94.4 |
| **2015** | | | | | | | | | | |
| **Axis** | **1** | **2** | **3** | **4** | **5** | **6** | **7** | **8** | **9** | **10** |
| **Eigenvalue** | 0.18 | 0.14 | 0.12 | 0.11 | 0.10 | 0.09 | 0.08 | 0.08 | 0.07 | 0.07 |
| **Variance (%)** | NA | NA | NA | NA | NA | NA | NA | NA | NA | NA |
| **Adjusted variance (%)** | 33.5 | 16.8 | 11.6 | 9.9 | 7.1 | 4.7 | 3.3 | 3.2 | 2.5 | 2.3 |
| **Cumulated adjusted variance (%)** | 33.5 | 50.3 | 61.9 | 71.8 | 78.9 | 83.6 | 86.9 | 90.1 | 92.6 | 94.9 |

*Table F: Contributive modalities to the first three axes. 1910*


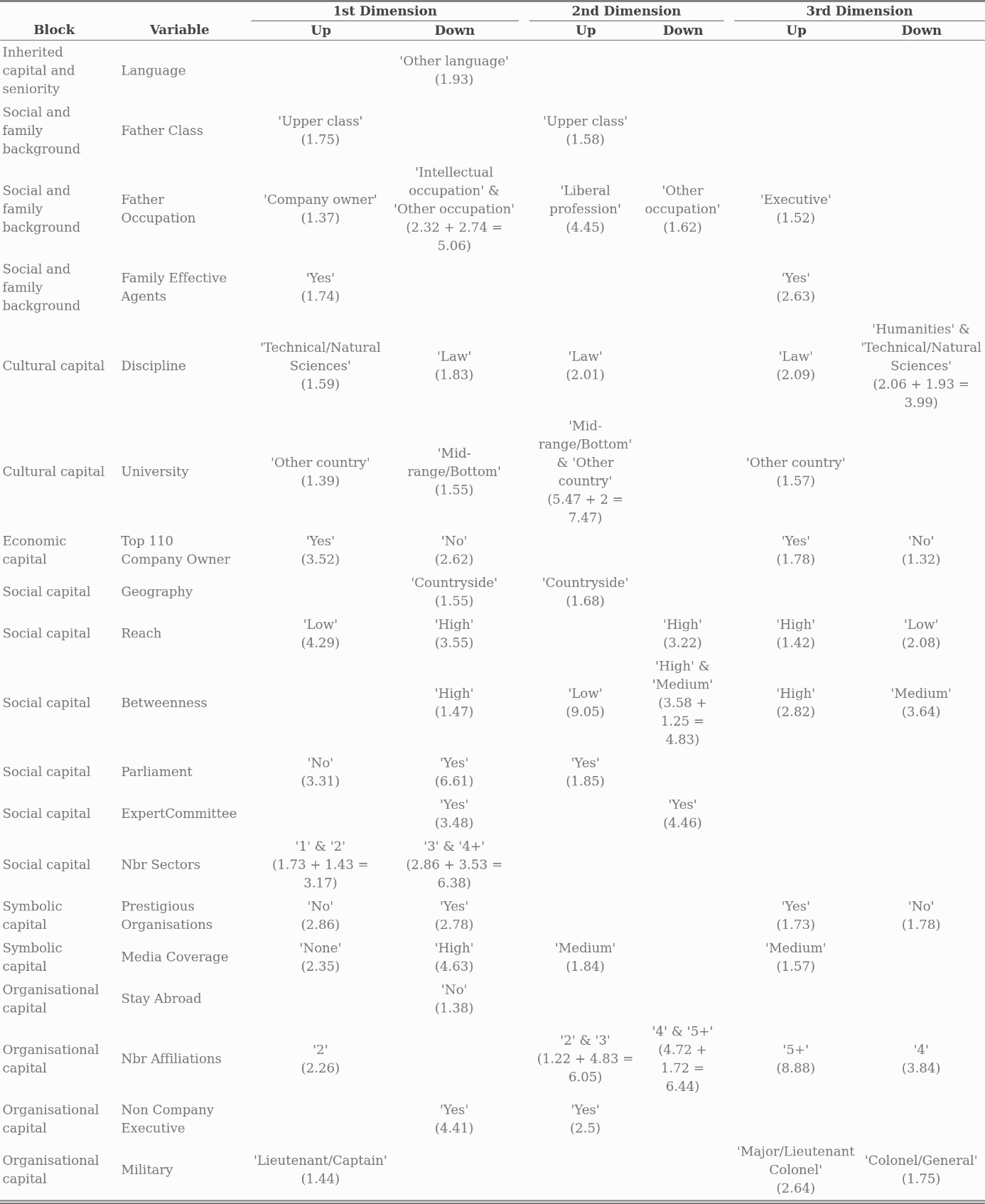


*Table G: Contributive modalities to the first three axes. 1937*

*
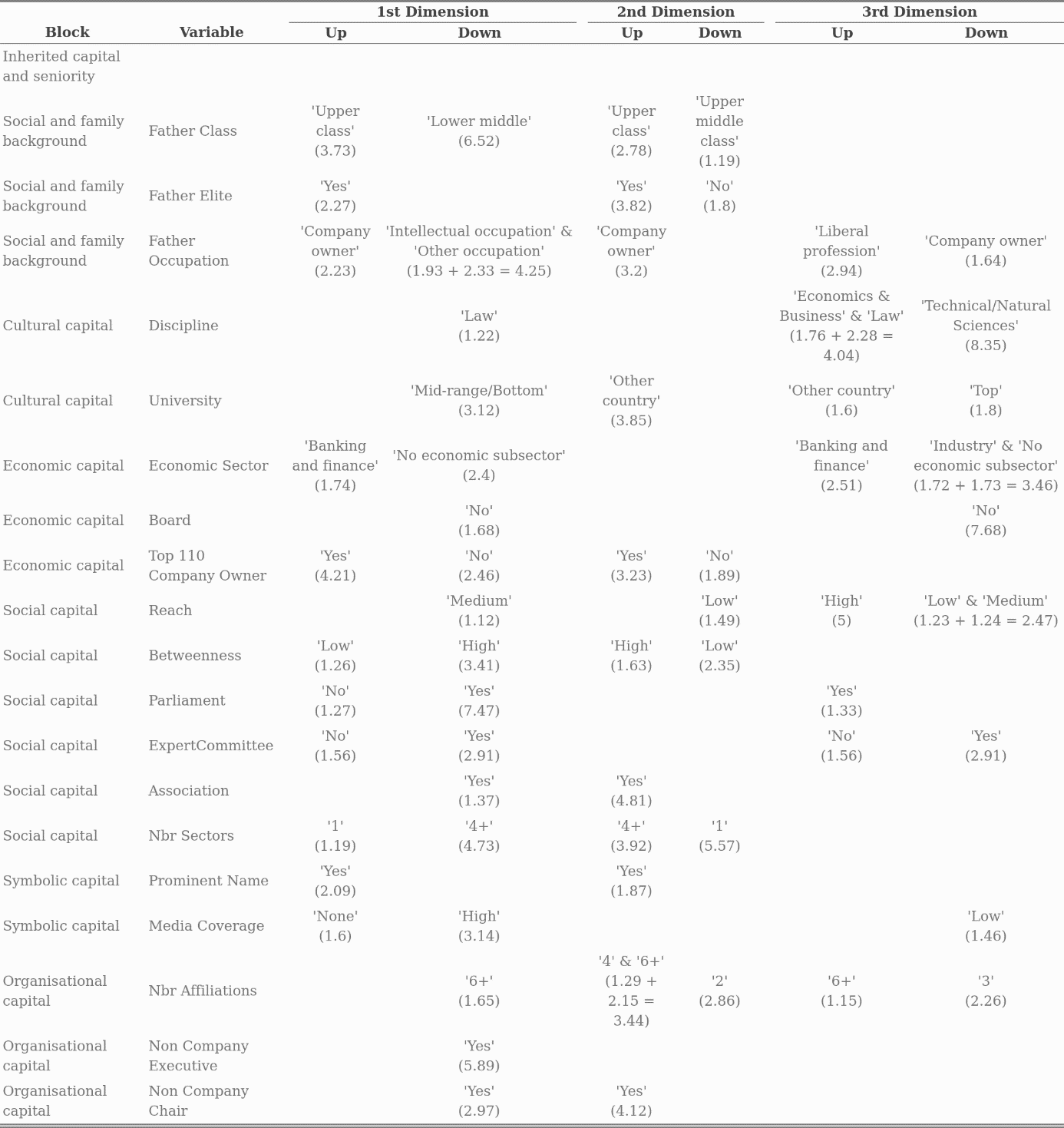
*

*Table H: Contributive modalities to the first three axes. 1957*

*
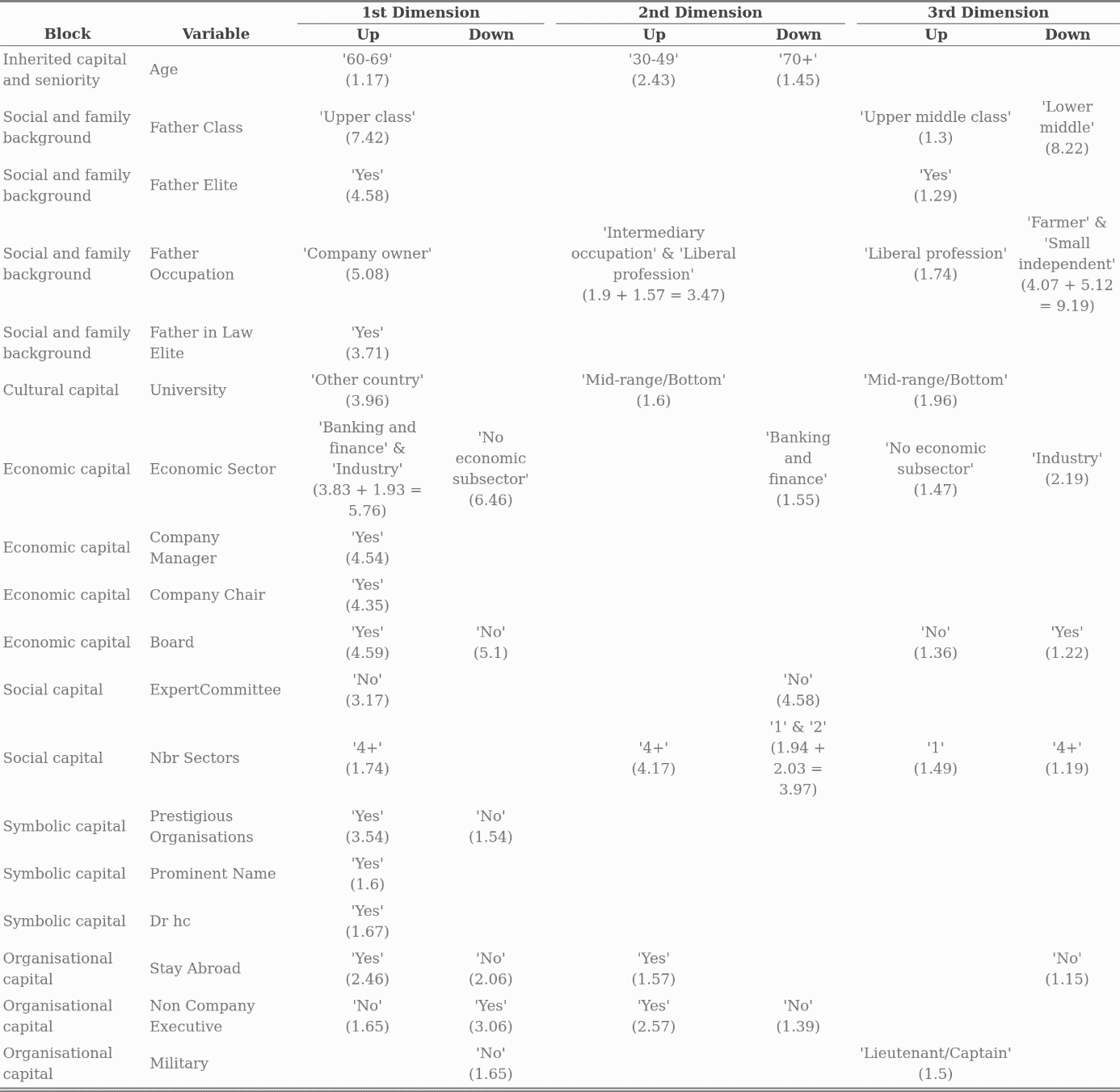
*

*Table I: Contributive modalities to the first three axes. 1980*

*
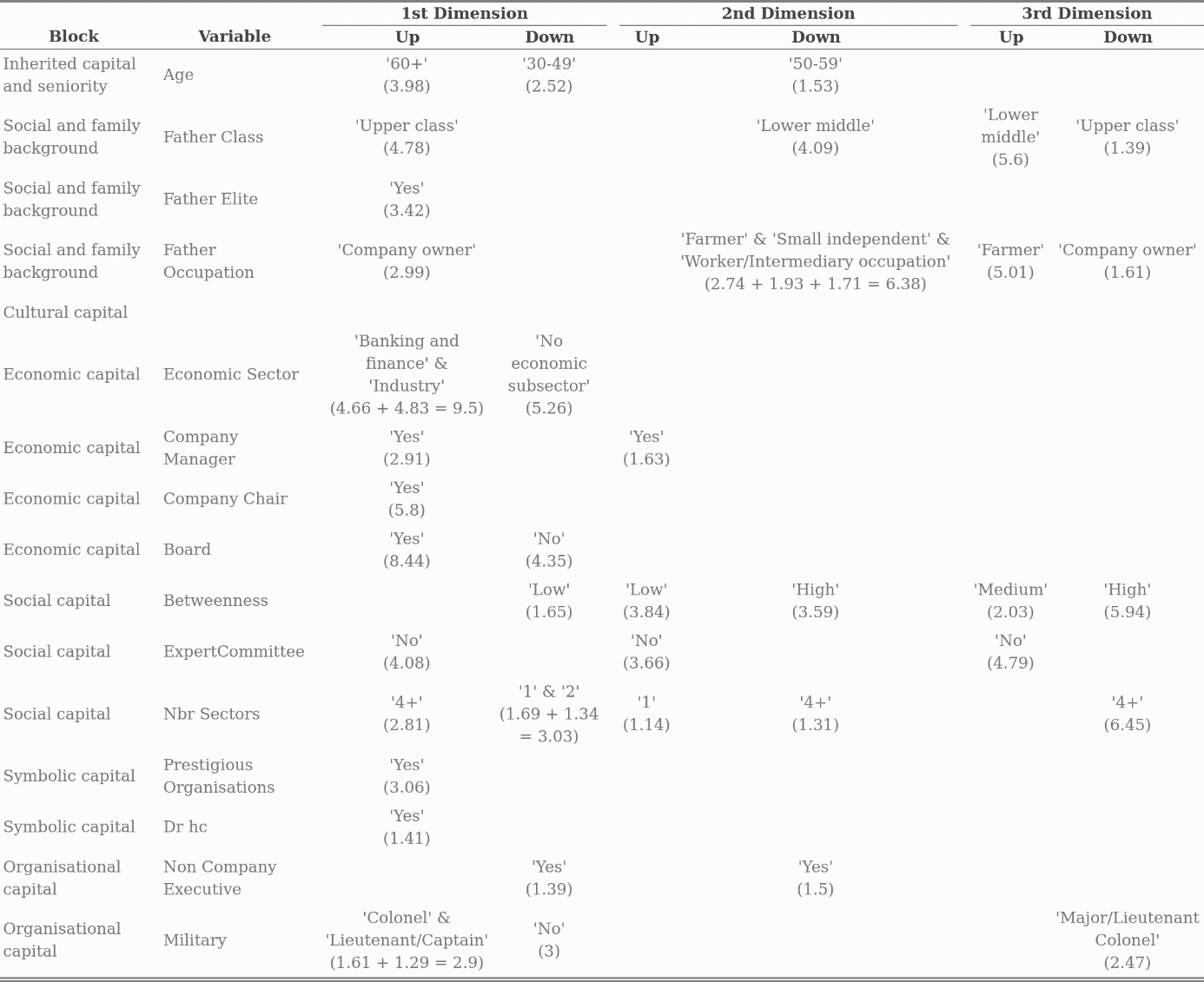
*

*Table J: Contributive modalities to the first three axes.2000*

*
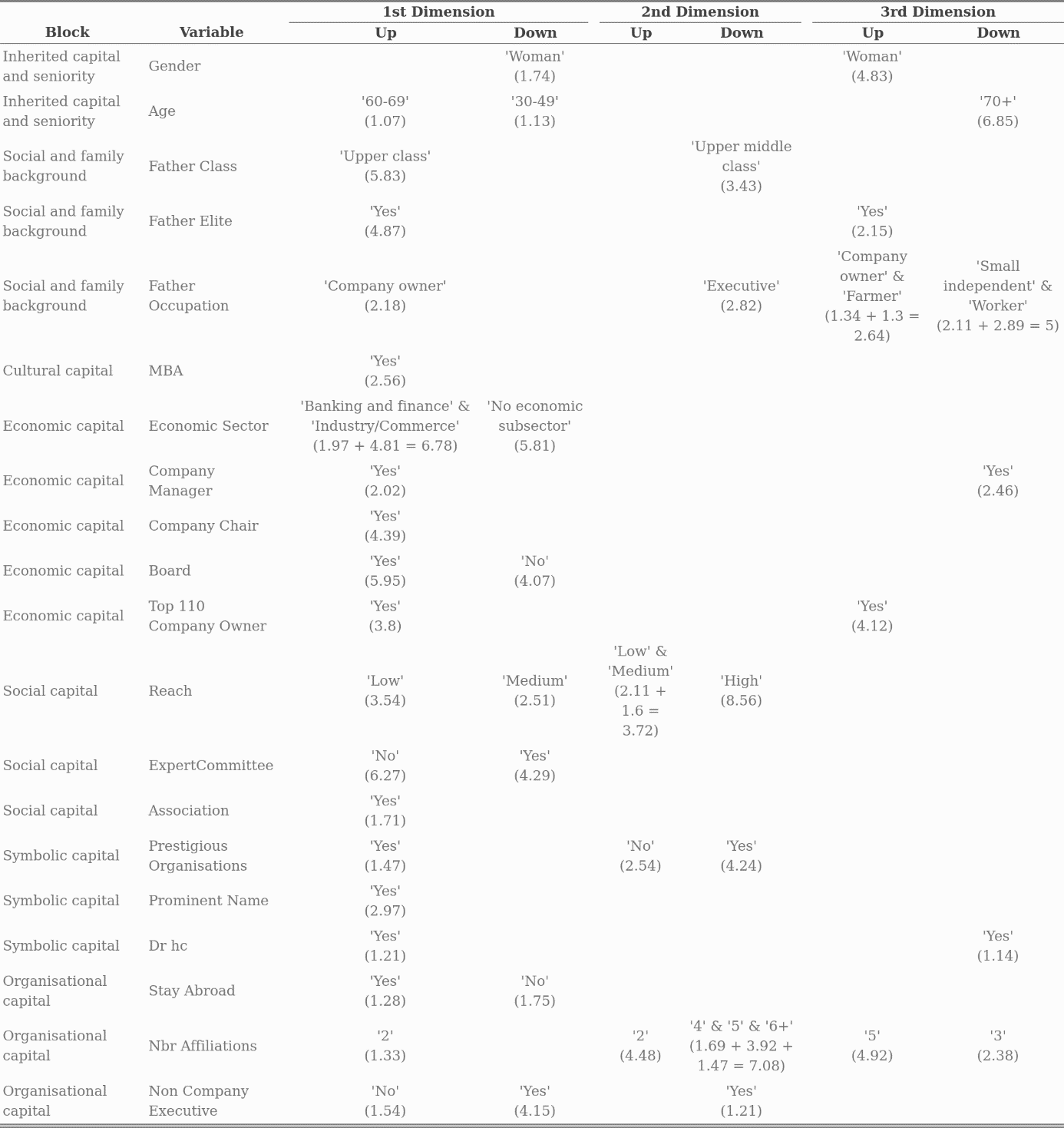
*

*Table K: Contributive modalities to the first three axes. 2015*

*
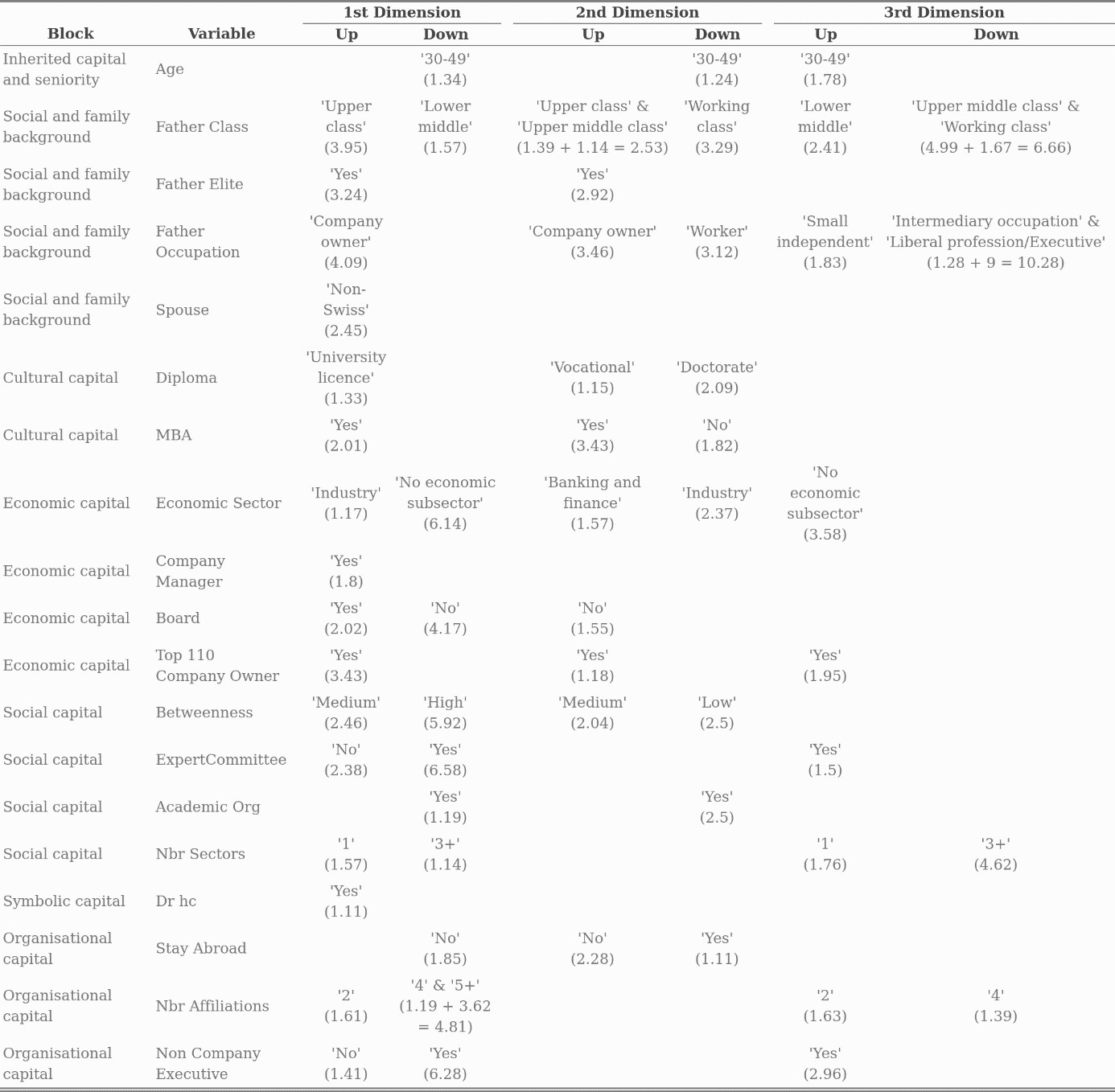
*

*Figure A: Contributive modalities to the first two axes. 1910*

*
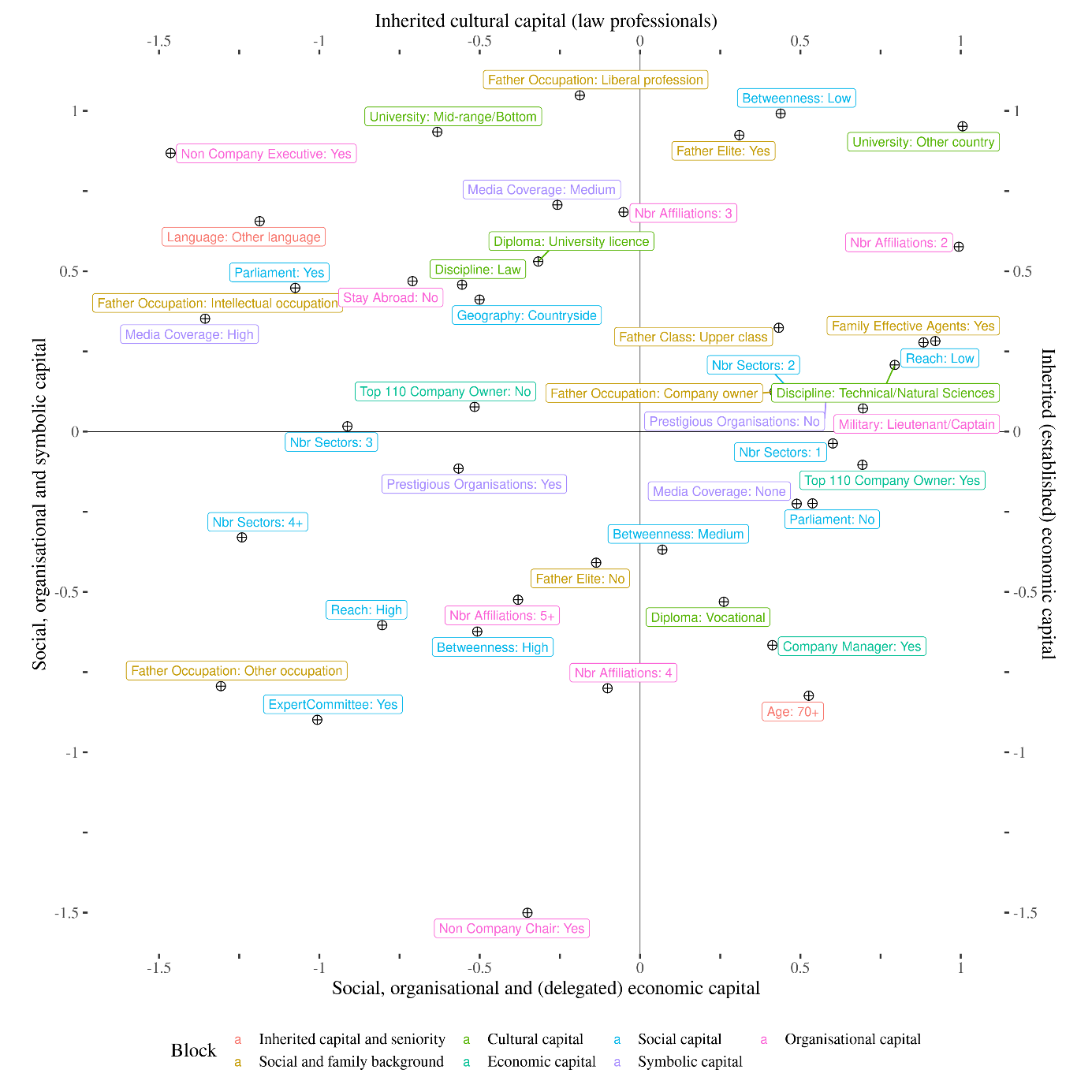
*

*Figure B: Contributive modalities to the first two axes. 1937*

*
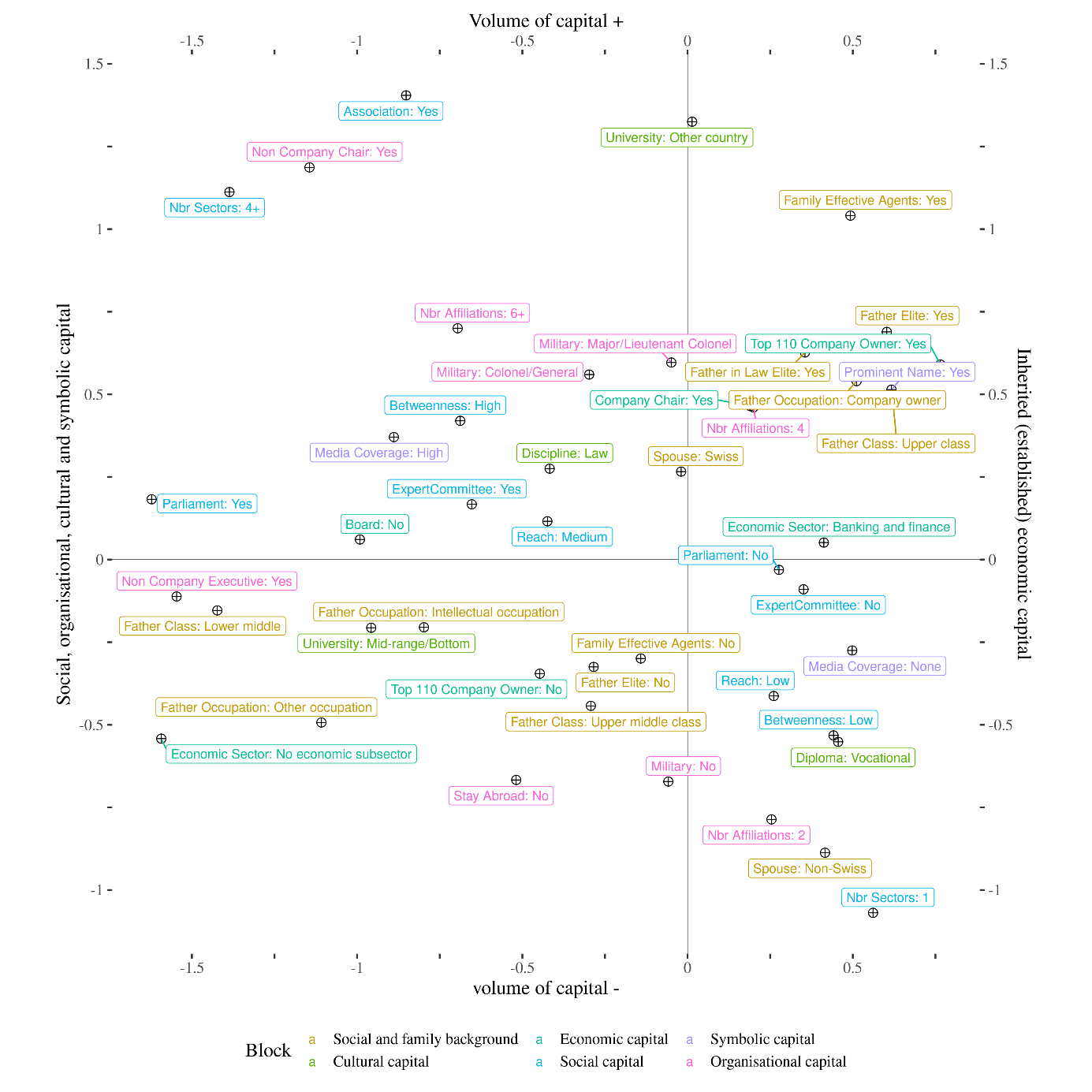
*

*Figure C: Contributive modalities to the first two axes. 1957*

*
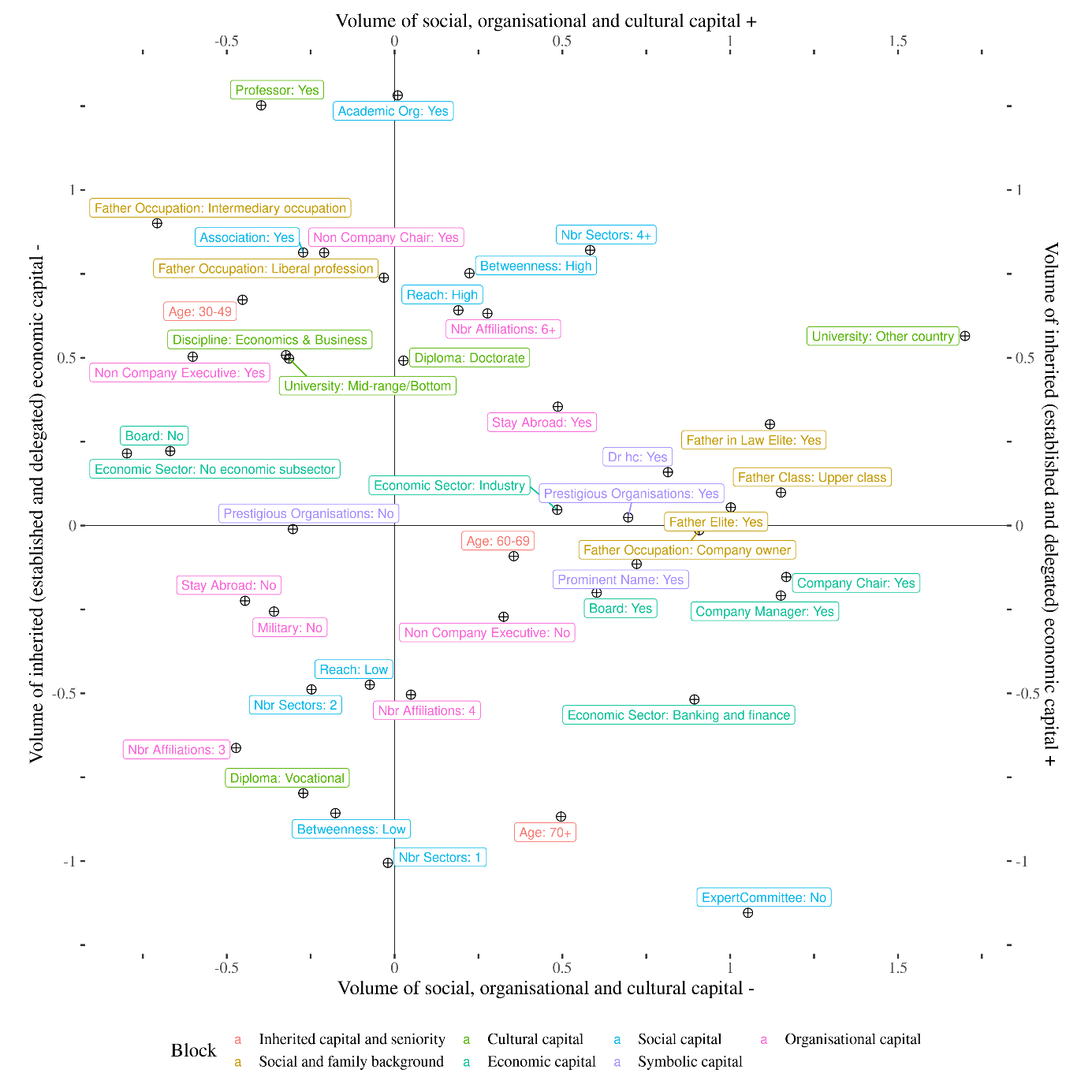
*

*Figure D: Contributive modalities to the first two axes. 1980*

*
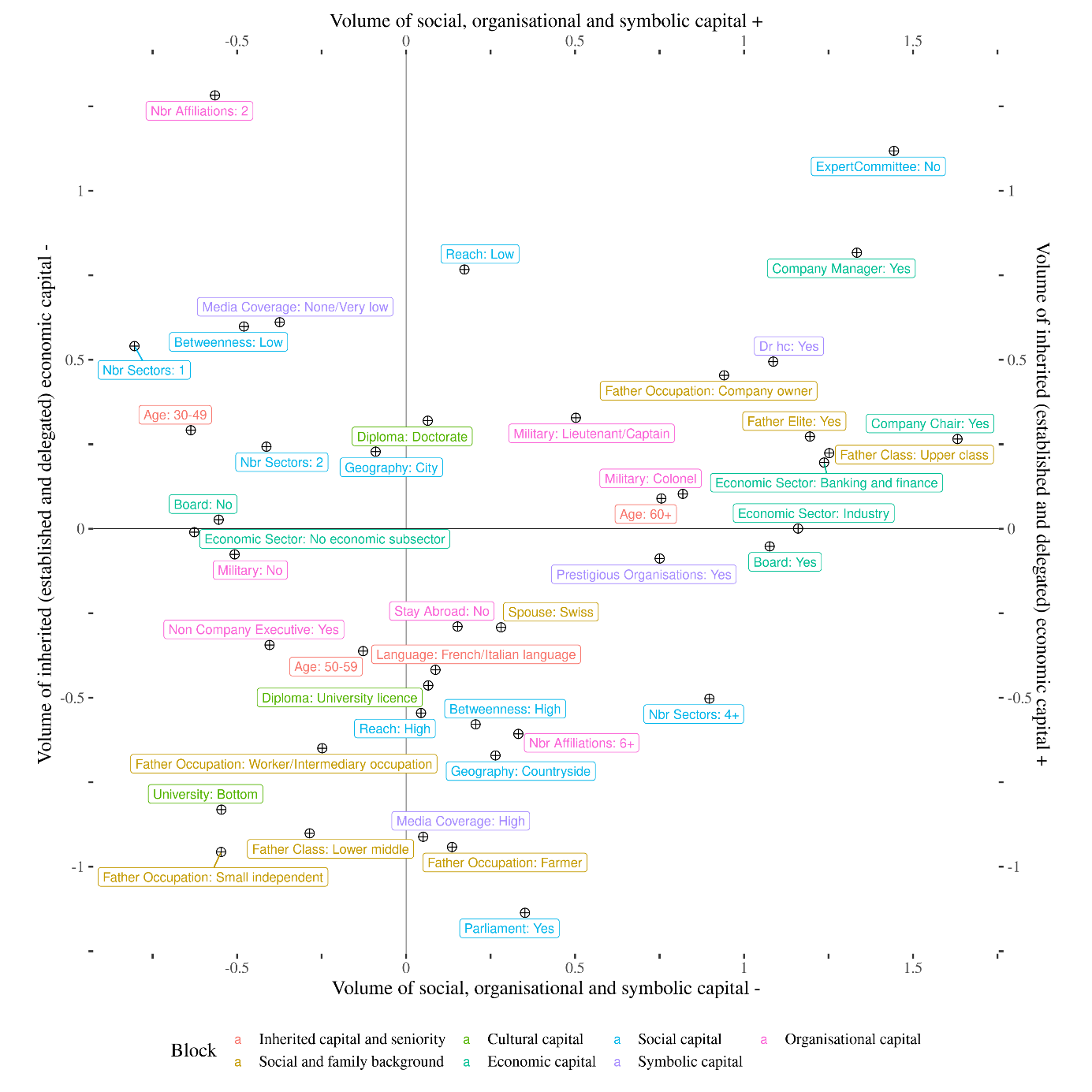
*

*Figure E: Contributive modalities to the first two axes. 2000*

*
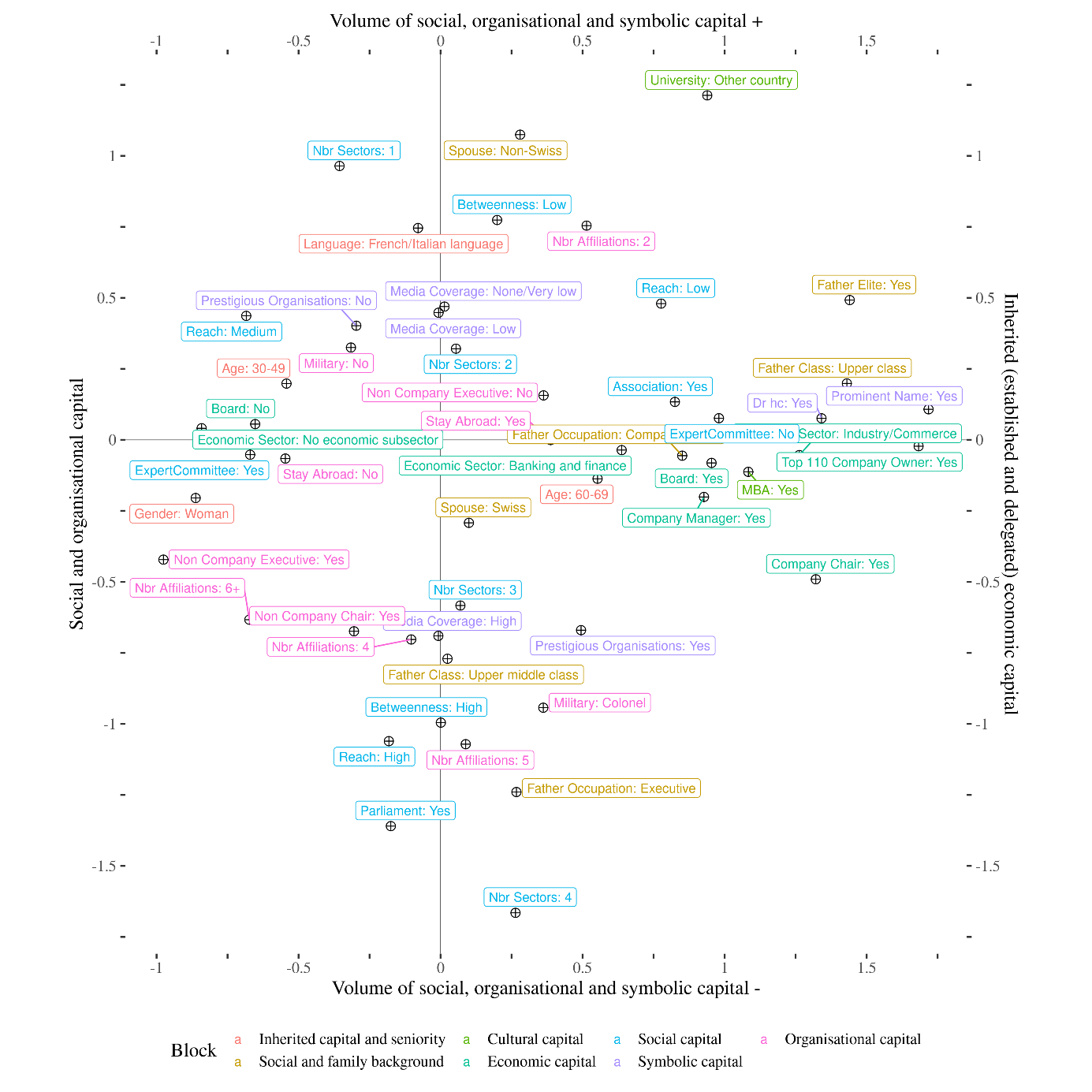
*

*Figure F: Contributive modalities to the first two axes. 2015*

*
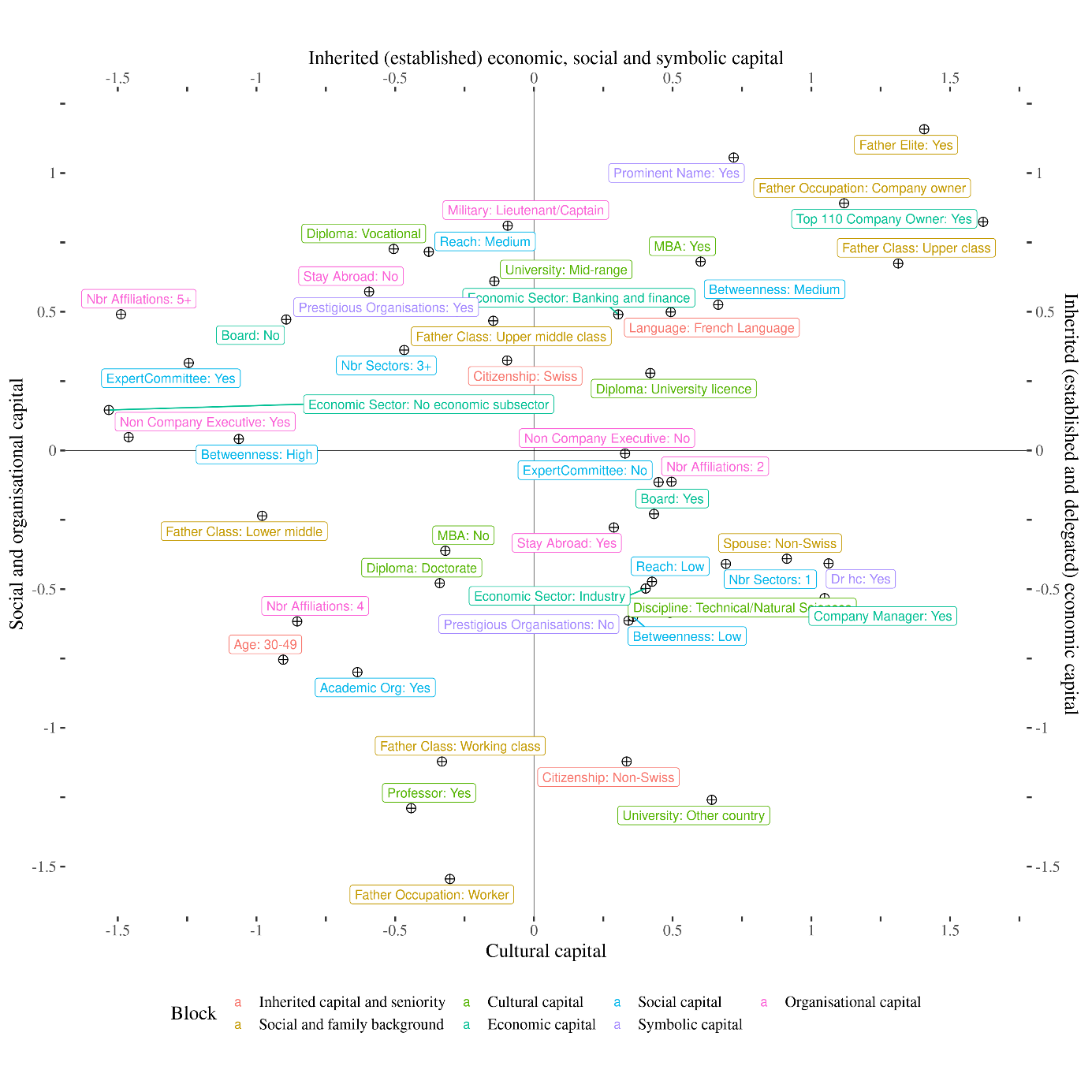
*

*Figure G: Sector (supplementary variable). 1910-2015*

*
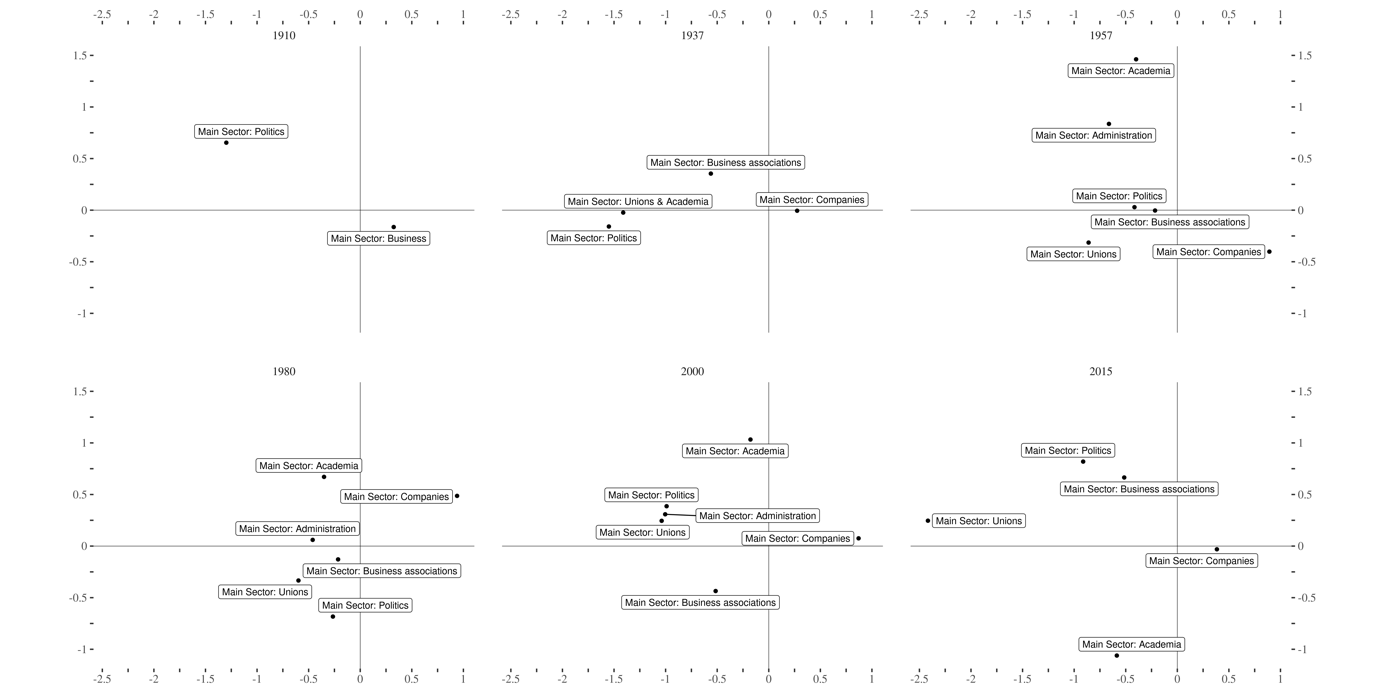
*

**References**

Benz P, Araujo P, Legentilhomme G, et al. (2024) The Swiss Patrician Families between Decline and Persistence: Power Positions and Kinship Ties (1890–1957). Social Science History 48: 331–360.

Bühlmann F, David T and Mach A (2013) Cosmopolitan Capital and the Internationalization of the Field of Business Elites: Evidence from the Swiss Case. *Cultural Sociology* 7(2): 211–229.

David T, Mach A, Lüpold M, et al. (2015) *De La “Forteresse Des Alpes” à La Valeur Actionnariale. Histoire de La Gouvernance d’entreprise Suisse (1880-2010)*. Zurich and Geneva: Seismo.

Mach A, Araujo P, Benz P, et al. (eds.) (2024) *Élites et Pouvoir Dans Les Grandes Villes Suisses (1890-2020)*. Neuchâtel: Editions Alphil.

1. See: David et al. (2015), particularly the appendix pp. 473-501, for the complete methodology of the choice of the companies and their list. [↑](#footnote-ref-1)
